# Wolframin-1–expressing neurons in the entorhinal cortex propagate tau to CA1 neurons and impair hippocampal memory in mice

**DOI:** 10.1126/scitranslmed.abe8455

**Published:** 2021-09-15

**Authors:** Jean-Christophe Delpech, Dhruba Pathak, Merina Varghese, Srinidhi Venkatesan Kalavai, Emma C. Hays, Patrick R. Hof, W. Evan Johnson, Seiko Ikezu, Maria Medalla, Jennifer I. Luebke, Tsuneya Ikezu

**Affiliations:** 1 Department of Pharmacology and Experimental Therapeutics, Boston University School of Medicine, Boston, MA, USA.; 2 University of Bordeaux, INRAE, Bordeaux INP, NutriNeuro, UMR 1286, F-33000 Bordeaux, France.; 3 Anatomy and Neurobiology, Boston University School of Medicine, Boston, MA, USA.; 4 Nash Family Department of Neuroscience, Friedman Brain Institute, and Ronald C. Loeb Center for Alzheimer’s Disease, Icahn School of Medicine at Mount Sinai, New York, NY, USA.; 5 Computational Biomedicine, Boston University School of Medicine, Boston, MA, USA.; 6 Center for Systems Neuroscience, Boston University, Boston, MA, USA.; 7 Department of Neuroscience, Mayo Clinic, Jacksonville, FL, USA.

## Abstract

Abnormally phosphorylated tau, an early neuropathologic marker of Alzheimer’s disease (AD), first occurs in the brain’s entorhinal cortex layer II (ECII) and then spreads to the CA1 field of the hippocampus. Animal models of tau propagation aiming to recapitulate this phenomenon mostly show tau transfer from ECII stellate neurons to the dentate gyrus, but tau pathology in the dentate gyrus does not appear until advanced stages of AD. Wolframin-1–expressing (Wfs1^+^) pyramidal neurons have been shown functionally to modulate hippocampal CA1 neurons in mice. Here, we report that Wfs1^+^ pyramidal neurons are conserved in the ECII of postmortem human brain tissue and that Wfs1 colocalized with abnormally phosphorylated tau in brains from individuals with early AD. Wfs1^+^ neuron–specific expression of human P301L mutant tau in mouse ECII resulted in transfer of tau to hippocampal CA1 pyramidal neurons, suggesting spread of tau pathology as observed in the early Braak stages of AD. In mice expressing human mutant tau specifically in the ECII brain region, electrophysiological recordings of CA1 pyramidal neurons showed reduced excitability. Multielectrode array recordings of optogenetically stimulated Wfs1^+^ ECII axons resulted in reduced CA1 neuronal firing. Chemogenetic activation of CA1 pyramidal neurons showed a reduction in c-fos^+^ cells in the CA1. Last, a fear conditioning task revealed deficits in trace and contextual memory in mice overexpressing human mutant tau in the ECII. This work demonstrates tau transfer from the ECII to CA1 in mouse brain and provides an early Braak stage preclinical model of AD.

## One-sentence summary

Wolframin-1–positive neurons in the entorhinal cortex of mouse brain propagate tau to the hippocampal CA1 region resulting in memory impairment.

**Editor’s Summary:**

**A neuronal highway for tau**

Abnormally phosphorylated tau has been observed in the entorhinal cortex layer II (ECII) of the brain and from there spreads to the hippocampal CA1 region during the early stages of Alzheimer’s disease. Here, Delpech *et al.* develop an animal model that recapitulates this early tau spreading pathology. They show that expressing human mutant tau specifically in Wolframin-1–positive neurons of the ECII of mouse brain resulted in transfer of tau specifically to the hippocampal CA1 area. This was accompanied by memory impairment and reduced activity of CA1 pyramidal neurons, suggesting that this neuronal pathway should be investigated further in Alzheimer’s disease.

## INTRODUCTION

In Alzheimer’s disease (AD), neurofibrillary tangles first accumulate in the transentorhinal and entorhinal cortex that projects to hippocampal subregions in the brain (1, 2). Phosphorylated tau (p-tau) first appears in entorhinal cortex layer II (ECII) and then is found in pyramidal neurons of the CA1 field of the hippocampus during Braak stage II of AD (fig. S1A) (3). Tau spread is thought to take place among synaptically connected brain regions; however, the pathway that connects ECII to CA1 involved in this early tau spreading remains unknown. ECII neurons are heterogeneous across species, showing two different chemical and morphological phenotypes: Reelin^+^ multipolar stellate neurons and calbindin^+^ pyramidal neurons (4–7). Reelin^+^ stellate neurons are at the origin of the perforant pathway connecting the entorhinal cortex to the dentate gyrus; Wolframin-1^+^ pyramidal neurons (Wfs1^+^), so-called island cells in rodents, coincide with calbindin^+^ neuron clusters in the medial ECII (MECII). In contrast to rodents, MECII in human brain is largely composed of multipolar reelin^+^ cells that form island-like clusters (1); calbindin^+^ cell clusters, on the other hand, are most prominent in the caudal pole of ECII in human brain (4, 7) where p-tau accumulates in the very early Braak stages of AD. This prompted us to investigate the role of Wfs1^+^ neurons in tau propagation in mouse brain.

Recently, Wfs1^+^ projections from ECII neurons to CA1 were proposed to form the temporoammonic pathway, which is involved in the modulation of temporal associative memory (fig. S1B) (8). The mechanism and nature of tau propagation from ECII to the hippocampal CA1 brain region remains unclear, and current animal models do not recapitulate predominant and restricted tau propagation from ECII to CA1. Tau propagation animal models show p-tau accumulation principally in the dentate gyrus due to the overexpression of mutated tau in reelin^+^ neurons in transactivator neuropsin-tTA transgenic mice (9–11) or nonspecific expression by adeno-associated virus (AAV) (12, 13). As tau accumulation in the dentate gyrus does not appear until later AD stages, identifying the tau transfer pathway from ECII to CA1 could advance our understanding of early AD pathology. The recent description of the Wfs1 temporoammonic pathway has opened an avenue for the investigation of tau propagation during early AD in rodent models. We hypothesized that tau is propagated along the Wfs1^+^ ECII-CA1 pathway, inducing disconnection of this circuit and contributing to the memory deficits observed in early AD stages. Here, we used Wfs1-Cre transgenic mice and a Flex-AAV viral vector system to express human P301L mutant tau specifically in Wfs1^+^ neurons in the MECII brain region (ECII-CA1 tau mice). We examined the tau propagation pathway, specifically focusing on the synaptic connections between entorhinal cortex axonal terminals and CA1 dendrites in the stratum lacunosum in the hippocampal CA1 region. We assessed CA1 pyramidal neuronal function in ECII-CA1 tau mice using three different measures and analyzed the effect of tau propagation in CA1 on associative learning memory using a fear conditioning behavioral test.

## RESULTS

### Mouse ECII Wfs1^+^ neurons transfer tau specifically to CA1 pyramidal neurons

**Fig. 1.**
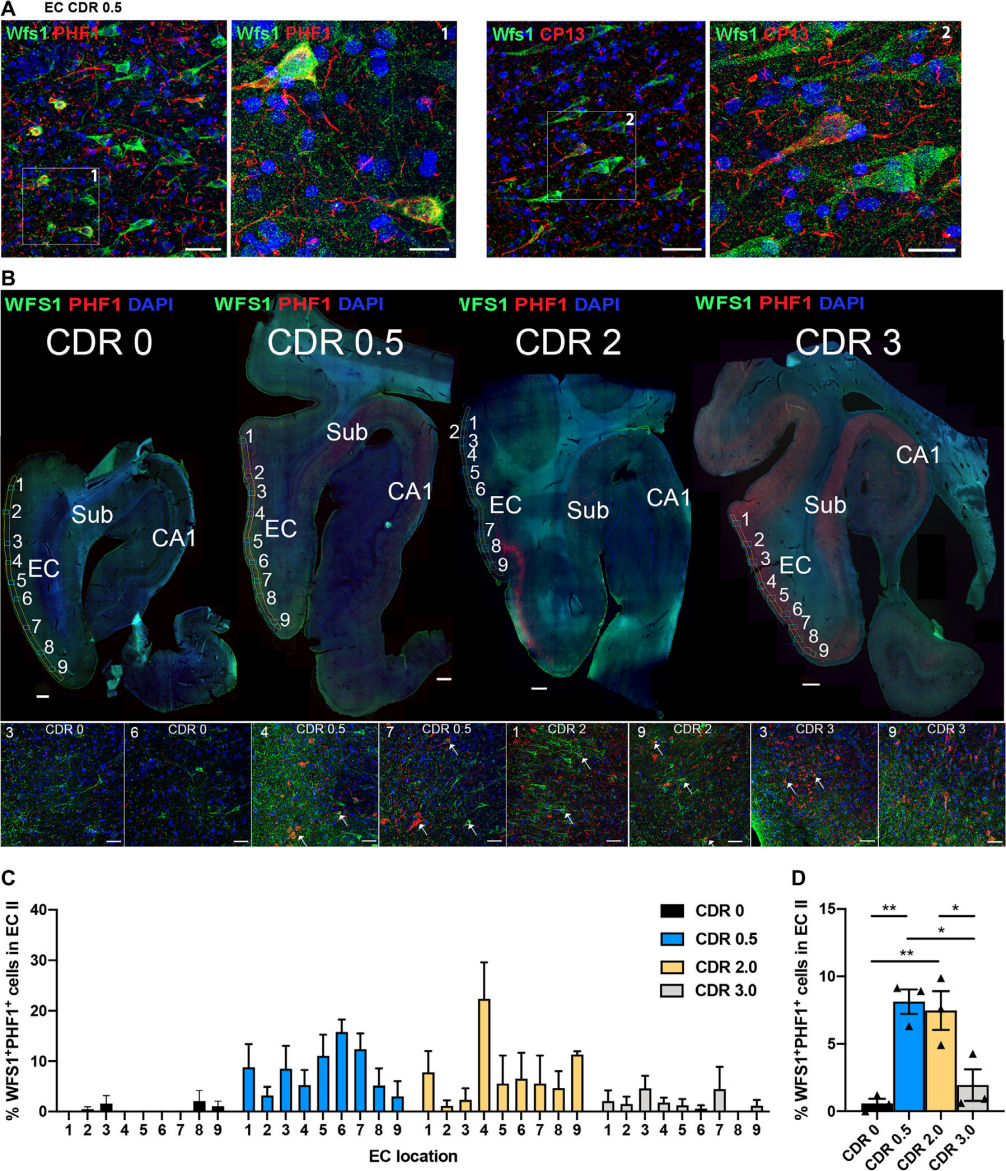
PHF1 antibody binding to WFS1^+^ neurons in human AD postmortem brain samples. (**A**) Shown is a horizontal section of human postmortem brain tissue from a patient with early stage AD [Clinical Dementia Rating (CDR) score 0.5, Braak stage III; table S1] showing the entorhinal cortex (EC) and WFS1^+^ neurons (green) in layers II and III. Phosphorylated tau (p-tau), detected by PHF1 antibody (left, red) or CP13 antibody (right, red), is present in a few WFS1^+^ neurons. Scale bars, 50 μm for 20× images in large panels and 20 μm for enlarged 63× images. (**B**) Shown are horizontal sections of postmortem human brain tissue from four patients (CDR 0, Braak Stage I, 85-year-old male; CDR 0.5, Braak stage III, 85-year-old male; CDR 2, Braak stage III, 88-year-old female; CDR 3, Braak stage V, 85-year-old male), with the EC and hippocampal CA1 areas indicated. Sections were immunostained with anti-WFS1 antibody (green) and PHF1 antibody (red). Sub, subiculum. The numbers 1 to 9 reflect the EC images used for quantification. Insets (bottom row) represent two examples of EC images per CDR group, and white arrows in insets indicate WFS1-PHF1 colocalization. Scale bars, 1000 μm for top panel images and 50 μm for bottom row inset images. (**C**) Shown is the distribution pattern of WFS1^+^PHF1^+^ neurons across the EC region of postmortem brain samples from 3 controls and 9 patients at various stages of AD with CDRs of 0, 0.5, 2, or 3 (12 patients total, *n* = 3 per CDR group; tables S1 and S2). (**D**) Quantification of WFS1^+^PHF1^+^ neurons in the EC upper layer of postmortem brain samples from 3 controls and 9 patients at various stages of AD with CDRs of 0, 0.5, 2, or 3, 12 patients total, *n* = 3 per CDR group [one-way ANOVA, *F*_(3,8)_ = 13.43 **P < 0.01; Tukey’s post hoc, test *P < 0.05 and **P < 0.01; tables S1 and S2].

Calbindin^+^ neurons have been previously reported in the upper layers of human entorhinal cortex, although positivity for Wfs1 in those neurons has not been investigated (7). We confirmed the existence of Wfs1^+^ pyramidal neurons in ECII of 12 postmortem human brain samples (Fig. 1A). We observed that Wfs1^+^ neurons in ECII also partially colocalized with p-tau (PHF1 for pSer^396^/pSer^404^ tau and CP13 for pS^202^ tau epitopes), indicating the presence of p-tau in Wfs1^+^ ECII neurons (Fig. 1A). We then quantified the number of Wfs1^+^ neurons in the ECII and examined their colocalization with tau using PHF1 antibody in 12 postmortem human brain samples from individuals with clinical dementia rating (CDR) scores ranging from 0 to 3 (table S1). Our quantification indicated that Wfs1^+^ neurons represented 6.5% (CDR 0), 6.1% (CDR 0.5), 2.5% (CDR 2), and 2.7% (CDR 3) of the 4′,6-diamidino-2-phenylindole–positive (DAPI^+^) cells in the ECII of human brain (Fig. 1B). We further found that 8.1 and 7.5% of ECII Wfs1^+^ neurons were PHF1^+^ for p-tau in CDR 0.5 and 2 brain samples, but 0.6 and 1.9% in CDR 0 and 3 brain samples, respectively (Fig. 1, C and D). These data suggested that p-tau accumulated in ECII Wfs1^+^ neurons during the early stages of cognitive decline in individuals with AD.

**Fig. 2.**
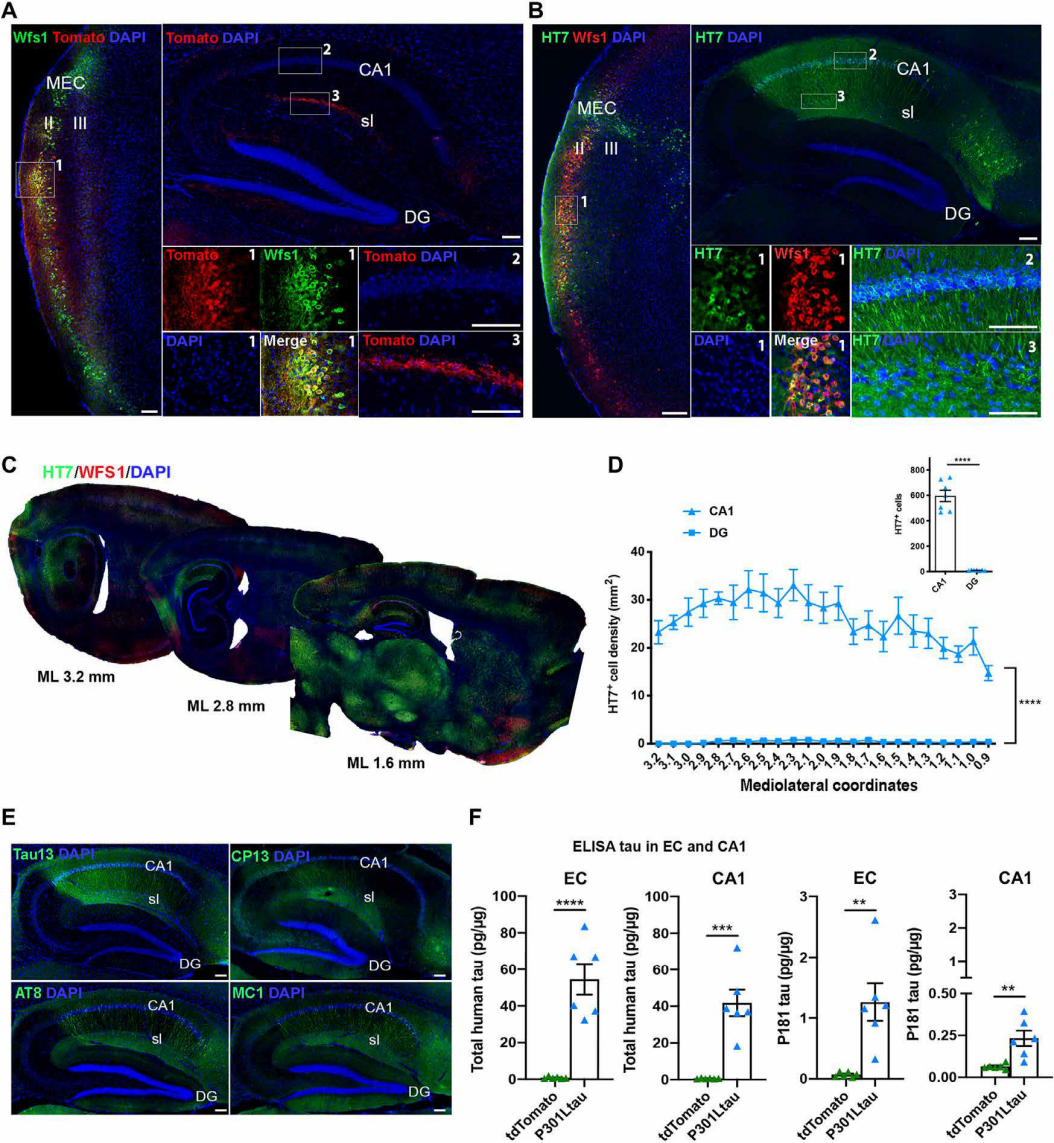
Wfs1^+^ neurons in mouse EC layer II propagate human tau to hippocampal CA1. AAV2/6-Flex-tdTomato as a control or AAV2/6-Flex-P301L human mutant tau was injected unilaterally into the medial EC (MEC) of Wfs1-Cre mice. (**A**) Shown is a representative parasagittal section of the MEC and hippocampal CA1 area of a Wfs1-Cre mouse brain injected with AAV2/6-Flex-tdTomato as a control, immunostained with anti-RFP antibody (red) and anti-Wfs1 antibody (green). Scale bars, 100 μm (*n* = 10 sections from 10 animals). (**B** and **C**) Shown is a representative parasagittal section of MEC and the hippocampal CA1 area of a Wfs1-Cre mouse brain injected with AAV2/6-Flex-P301L human mutant tau. Sections were immunostained with anti-human tau HT7 antibody (green) and anti-Wfs1 antibody (red) 4 weeks after MEC injection. Scale bars, 100 μm (*n* = 26 sections from 7 animals). (**D**) Quantification of HT7^+^ neurons in the hippocampal CA1 area and dentate gyrus (DG) of Wfs1-Cre mouse brain injected with AAV2/6-Flex-P301L human mutant tau and immunostained with anti-human tau HT7 antibody at 4 weeks after MEC injection [two-way ANOVA on repeated measures, brain region tau effect *F*_(1,12)_ = 173.9 ****P < 0.0001; average: unpaired *t* test, *t* = 13.19, ****P < 0.0001, *n* = 7 animals]. (**E**) Shown is a representative parasagittal section of the hippocampal CA1 area of a Wfs1-Cre mouse brain injected with AAV2/6-Flex-P301L mutant tau. Sections were immunostained with anti-Tau13 antibody (green) or anti-MC1 antibody (misfolded tau, green) or CP13 antibody (pSer^202^ tau, green) or AT8 antibody (pSer^202^/pThr^205^ tau, green) 4 weeks after MEC injection. Scale bars, 100 μm (*n* = 5 sections from 4 animals). (**F**) Quantification of human tau and pThr^181^ tau in EC and CA1-enriched hippocampal tissue of Wfs1-Cre mouse brain 4 weeks after MEC injection of AAV2/6-Flex-P301L mutant tau (unpaired *t* test, total tau EC: *t* = 6.335, CA1: *t* = 5.681, P181tau EC: *t* = 3.851, CA1: *t* = 3.639; **P < 0.01, ***P < 0.001, and ****P < 0.0001, *n* = 6 mice per group; table S2).

To assess the connection from ECII Wfs1^+^ neurons to CA1 pyramidal neurons, we used Wfs1-Cre transgenic mice expressing Cre recombinase under the *Wfs1* promotor (8) and injected Cre-inducible AAV expressing tdTomato (AAV2/6-Flex-tdTomato) into the MEC of Wfs1-Cre mice (fig. S2A). We found restricted expression of tdTomato in ECII Wfs1^+^ neurons in the Wfs1-Cre mice (Fig. 2A, left, box 1), whereas few CA1 pyramidal neurons were tdTomato^+^ (Fig. 2A, top right, box 2). Wfs1^+^ axons primarily projected to the stratum lacunosum of CA1 (Fig. 2A, top right, box 3), although other projections were also found in the subiculum and in the contralateral CA1 stratum lacunosum and entorhinal cortex (fig. S2, B and C). Stereotactic injections of Cre-inducible AAV expressing human P301L mutant tau (AAV2/6-Flex-P301L tau) into MEC of Wfs1-Cre mice showed restricted expression of human tau in Wfs1^+^ ECII neurons at 1 week after injection, visualized using human tau-specific HT7 antibody (fig. S3, A to C); there were few reelin^+^ HT7^+^ neurons in the ECII of mouse brain (fig. S3C) and very few HT7^+^ cells in the CA1 region (fig. S3B). Moreover, no HT7 antibody signal was detected 1 week after Cre-dependent human P301L mutant tau was injected into wild-type mice (fig. S4, A to C). At 4 weeks after injection, we observed a stronger HT7 signal in ECII Wfs1^+^ neurons (Fig. 2B, left, box 1) and a strong signal for HT7^+^ cells in the subiculum and CA1 including the stratum lacunosum and the pyramidal layer, but not in the dentate gyrus on the ipsilateral side (Fig. 2B, top right, boxes 2 and 3). Few HT7^+^ neurons were observed in the contralateral entorhinal cortex, stratum lacunosum, and CA1 pyramidal layer (fig. S5, A to C). As a control, we injected AAV2/6-SYN1-P301L mutant tau, which expressed human P301L mutant tau under the synapsin-1 promoter, into the same brain region of wild-type mice. We observed binding of HT7 antibody mainly to neurons of the ECII and ECIII, and their axonal projections could be traced to CA1 stratum lacunosum and the outer layer of the dentate gyrus (fig. S6, A to C), suggesting less regional specificity of tau propagation in wild-type mice. Quantification of the HT7^+^ neurons in the CA1 (mediolateral, +0.9 to +3.2 sagittal sections) confirmed preferential spread of mutant tau to the CA1 compared to the dentate gyrus (Fig. 2, C and D). In addition, we also observed a HT7^+^ signal in axons in the corpus callosum and stratum radiatum of the CA1 (fig. S7) and in the neuronal somata of the claustrum, subiculum, perirhinal cortex, visual cortex, parasubiculum, retrosplenial agranular cortex, retrosplenial granular cortex, magnocellular preoptic nucleus, rostral part of the thalamic nucleus, and substantia nigra pars compacta and pars reticulata (Fig. 2C and fig. S7). In our study, this was not due to the spread of AAV itself to the CA1, which only showed a limited amount of AAV-derived expression of the human *MAPT* mRNA or woodchuck hepatitis virus posttranscriptional regulatory element (*WPRE*) 3′ untranslated region (3′UTR) RNA as determined by in situ hybridization (fig. S8, A to C). Quantification of human *MAPT* mRNA expression in cells by in situ hybridization represented only 6.0 ± 0.8% of the HT7^+^ cells in the CA1 ipsilateral area of Wfs1-Cre mouse brains. There was no difference in the number of HT7^+^ cells in the CA1 or dentate gyrus regions between male and female Wfs1-Cre mice, leading us to combine data from both sexes (fig. S9, A and B).

We next validated the presence of human tau in mouse brain using the specific anti-tau antibody Tau13; we detected the accumulation of p-tau using antibodies CP13 (against pSer^202^ tau), AT8 (against pSer^202^/pThr^205^ tau), and MC1 (against misfolded tau) in mouse brain ECII (fig. S10, A and B), CA1, and subiculum (Fig. 2E). We also biochemically quantified the amount of human tau and p-tau in mouse entorhinal cortex, CA1 and frontal cortices by enzyme-linked immunosorbent assay (ELISA), confirming the accumulation of human tau and p-tau (pThr^181^ tau) in the entorhinal cortex and CA1 (Fig. 2F); we found only a very low amount of total tau and an absence of p-tau in the frontal cortices (fig. S11). Last, injecting human P301L mutant tau in dorsoventral coordinate 3.7 of the MEC of Wfs1-Cre mouse brain (more ventral, closer to the lateral entorhinal cortex) led to tau propagation to the dentate gyrus where Wfs1 was not expressed and, to a lesser extent, to CA1 compared to injection into dorsoventral coordinate 3.3 of the MEC (fig. S12).

### Morphological characterization and synaptic connections among ECII Wfs1^+^ neurons, stratum lacunosum interneurons, and CA1 pyramidal neurons

**Fig. 3.**
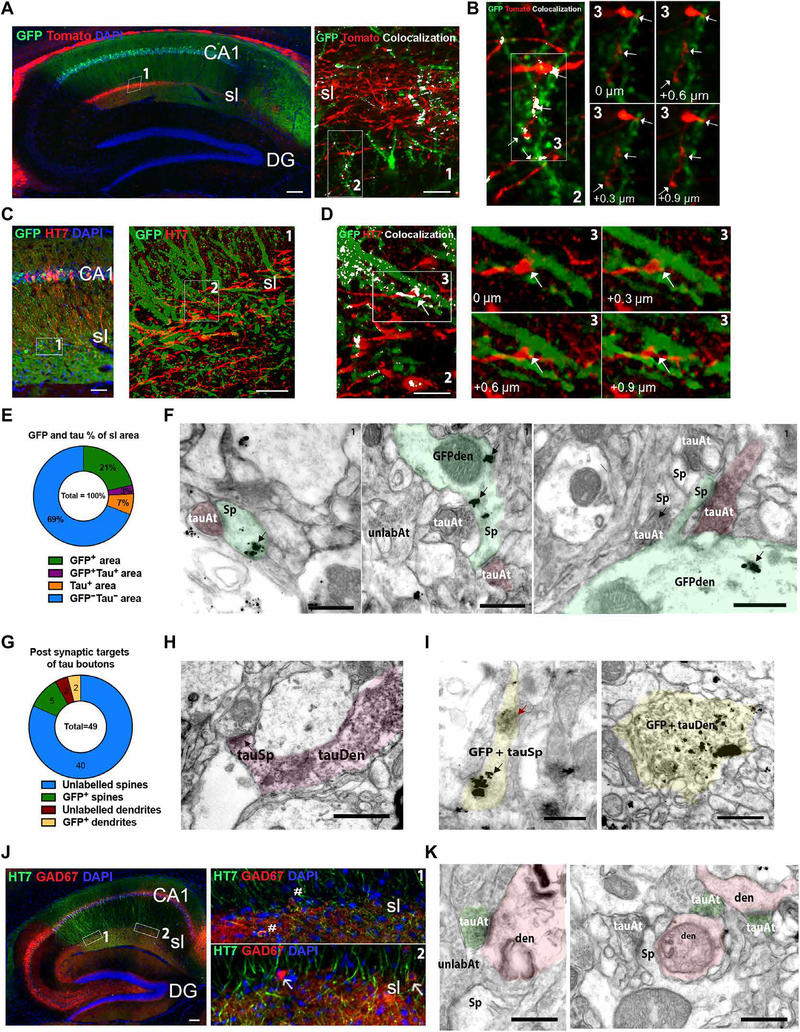
Wfs1^+^ neurons connected to stratum lucidum interneurons and CA1 pyramidal neurons propagate tau to mouse hippocampal CA1. (**A**) Left: Shown is a representative parasagittal section of the CA1 hippocampal region of Wfs1-Cre mouse brain injected unilaterally with AAV2/6-Flex-tdTomato (red) into the MEC and AAV2/6-Flex-GFP (green) injected unilaterally into the CA1. Brain sections were immunostained with anti-RFP/anti-GFP antibodies and were visualized by epifluorescence. Scale bar, 100 μm (*n* = 10 sections from 8 animals). (A) Right: High-resolution confocal microscopy of the CA1 stratum lacunosum (sl) (box 1; scale bar, 5 μm). (**B**) Left: Shown is a representative maximum projection of confocal image stacks showing colocalization of GFP and tdTomato immunostaining (white) in stratum lacunosum (sl). Scale bar, 5 μm. (B) Right: Single optical planes of the inset (left, box 3) show examples of appositions (white arrows) between GFP^+^ dendrites and tdTomato^+^ axons. (**C**) Left: Shown is a parasagittal section from the hippocampal region of Wfs1-Cre mouse brain injected with AAV2/6-Flex-P301L mutant tau unilaterally into the MEC and AAV2/6-Flex-GFP injected unilaterally into the hippocampal CA1 region. Sections were immunostained with anti-human tau HT7 antibody (red) and anti-GFP antibody (green) and were visualized by epifluorescence. Scale bar, 50 μm (*n* = 10 sections from 6 animals). (C) Right: High-resolution confocal microscopy image of the CA1 stratum lacunosum (sl) in box 1, left. Scale bar, 10 μm. (**D**) Left: Representative maximum projection of confocal image stacks of box 2 in Fig. 2C, right panel showing GFP (green) and tau (red) colocalization (white) in CA1 stratum laconosum. Scale bar, 5 μm. (D) Right: Single optical planes of the inset image (box 3, left) are shown in the four right panels and indicate examples of appositions (white arrows) between GFP^+^ spines and dendrites and tau^+^ axon terminals. (**E**) Quantification of the area labeled with GFP or tau or both in CA1 stratum lacunosum by confocal microscopy (two to five sections from 6 animals). (**F**) Electron micrographs of the Wfs1-Cre mouse brain CA1 stratum lacunosum showing examples of synaptic contacts between diaminobenzidine^+^ tau axon terminals (_tau_At, pink overlay) and GFP immunogold^+^ spines (Sp, black arrows, green overlay) on GFP immunogold ^+^ dendrites (GFPden). Note the appearance of the diaminobenzidine^+^ tau–labeled axons compared to unlabeled axon terminals (_unlab_At) nearby (*n* = 3 animals). (**G**) Quantification of the postsynaptic targets of tau boutons in stratum lacunosum measured by immunoelectron microscopy (pooled from *n* = 2 animals). (**H**) Representative electron micrograph of Wfs1-Cre mouse brain CA1 stratum lacunosum showing an example of a dendritic spine labeled with diaminobenzidine^+^ tau, emerging from a parent dendrite labeled with diaminobenzidine^+^ tau. Scale bar, 0.5 μm. (**I**) Electron micrographs of the CA1 stratum lacunosum showing an example of a dendritic spine (left) and a dendrite (right) double-labeled with both GFP immunogold (black arrow) and diaminobenzidine^+^ tau (red arrow). Scale bars, 0.5 μm. (**J**) Shown is a representative parasagittal section of the hippocampus of Wfs1-Cre mouse brain injected unilaterally with AAV2/6-Flex-P301L mutant tau into the MEC and immunostained by anti-human tau HT7 antibody (green) and anti-GAD67 antibody (red). White hash symbols indicate tau-positive GAD67 interneurons, and white arrows indicate tau-negative GAD67 interneurons. Scale bar, 100 μm (*n* = 6 sections from 4 animals). (**K**) Shown are electron micrographs of the Wfs1-Cre mouse brain CA1 stratum lacunosum region indicating an example of tau axon terminals (green) forming synapses on spiny dendrites (pink). Scale bars, 0.5 μm (table S2).

To understand how tau was transferred from ECII to CA1, we next determined in a direct or indirect manner whether Wfs1^+^ ECII axons projected to the dendrites of CA1 pyramidal neurons in mouse brain. We unilaterally injected AAV-Flex-tdTomato into MEC and AAV-Flex-GFP (green fluorescent protein) into CA1 brain regions of Wfs1-Cre mice (fig. S13A), which also expressed Cre in CA1 pyramidal neurons. Using epifluorescence and high-resolution confocal microscopy, we observed direct connections between Wfs1^+^ ECII axons and CA1 pyramidal dendrites through the apposition of expression of the two reporter genes in CA1 stratum lacunosum (Fig. 3A, left box 1, right box 2). Visualization of consecutive single optical planes and the three-dimensional (3D) reconstructed images showed clear apposition between GFP^+^ dendrites and tdTomato^+^ axons (Fig. 3B, box 3; fig. S14; and movie S1). This connection was further validated using Cre-inducible complementary rabies virus for monosynaptic retrograde tracing (fig. S15A). We demonstrated the presence of monosynaptic connections from CA1 pyramidal neurons to CA2, CA3, and stratum lacunosum neurons (fig. S15B) and to MEC layer III and some Wfs1^+^ ECII neurons (fig. S15C). These results confirmed the monosynaptic connection of ECII Wfs1^+^ neurons to CA1 pyramidal neurons. We next injected Wfs1-Cre mice with an AAV-Flex-GFP into CA1 and an AAV-Flex-P301L mutant tau into MEC regions. At 4 weeks after injection, we found colocalization of HT7 antibody with GFP in CA1 stratum lacunosum (Fig. 3C, left box 1 and right box 2). This was further revealed in adjacent optical planes and 3D images with clear apposition between GFP^+^ dendrites and HT7^+^ ECII axons, suggesting direct transfer of tau to CA1 dendrites from axonal terminals of ECII neurons (Fig. 3D, box 3; fig. S16; and movie S2). Quantification of GFP and tau colocalization in CA1 stratum lacunosum (Fig. 3E) showed colocalization coefficient values of 12.1 ± 2.7% between GFP^+^HT7^+^ and GFP^+^ areas and 29.3 ± 5.1% between GFP^+^HT7^+^ and HT7^+^ areas as determined by Mander’s coefficient. We next validated these findings using immunoelectron microscopy and detected the presence of HT7^+^ ECII axons making synaptic contacts with GFP^+^ spines of CA1 pyramidal dendrites in CA1 stratum lacunosum (Fig. 3F). We identified a total of 49 postsynaptic targets of tau^+^ boutons including 40 unlabeled spines, 5 GFP^+^ spines, 2 GFP^+^ dendrites, and 2 unlabeled dendrites (Fig. 3G), indicating the possible synaptic transfer of tau from presynaptic to postsynaptic elements. We also identified HT7^+^ spines emerging from HT7^+^ parent dendrites (Fig. 3H), as well as HT7^+^ GFP^+^ spines and dendrites (Fig. 3I). Knowing that ECII Wfs1^+^ neurons project to CA1 stratum lacunosum γ-aminobutyric acid (GABA)–ergic interneurons through the temporoammonic pathway, we also identified HT7^+^GAD67^+^ GABAergic interneurons in stratum lacunosum regions by confocal microscopy (Fig. 3J, boxes 1 and 2), and synaptic contacts between HT7^+^ ECII axons and morphologically identified dendrites of interneurons by immunoelectron microscopy (Fig. 3K).

### Impaired excitability of CA1 pyramidal neurons in ECII-CA1 tau mice

**Fig. 4.**
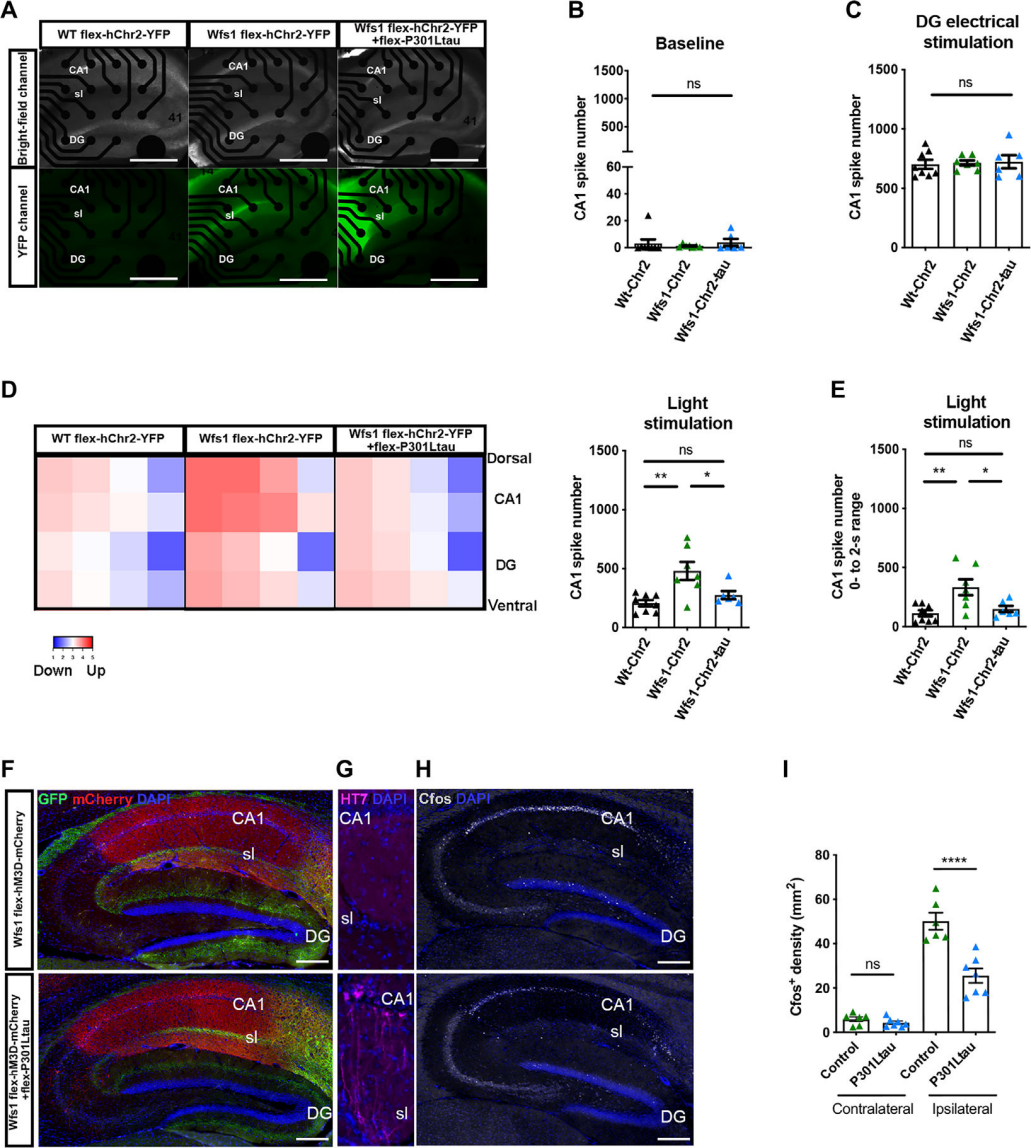
Tau propagation modulates mouse hippocampal CA1 response to optogenetic and pharmacologic stimulation. (**A**) Top row of photomicrographs show bright field images of wild-type (WT) or Wfs1-Cre mouse brain hippocampal slices 4 weeks after injection of a Cre-dependent channelrhodopsin-2 (Chr2) with or without Cre-dependent P301L mutant tau expression, positioned on a multielectrode array. Bottom row of photomicrographs show visualization of YFP in hippocampal slices from wild-type and Wfs1-Cre mouse brains 4 weeks after injection of a Cre-dependent Chr2 with or without Cre-dependent P301L mutant tau expression. Scale bars, 100 μm. (**B**) Shown is baseline neuronal activity in the hippocampal CA1 slices measured by multielectrode array 4 weeks after the injection of a Cre-dependent Chr2 with or without Cre-dependent P301L mutant tau expression in wild-type and Wfs1-Cre mouse brains [one-way ANOVA, *F*_(2,18)_ = 0.33, Wt-Chr2 group: *n* = 8 animals; Wfs1-Chr2 group: *n* = 7 animals; Wfs-Chr2-tau group: *n* = 6 animals]. (**C**) Shown is hippocampal CA1 slice neuronal activity measured by multielectrode array after dentate gyrus electrical stimulation, 4 weeks after the injection of a Cre-dependent Chr2 with or without Cre-dependent P301L mutant tau expression in wild-type and Wfs1-Cre mouse brains [one-way ANOVA, tau effect *F*_(2,18)_ = 0.08, Wt-Chr2 group: *n* = 8 animals; Wfs1-Chr2 group: *n* = 7 animals; Wfs-Chr2-tau group: *n* = 6 animals]. (**D**) Left: Shown is a heatmap representing the degree of hippocampal CA1 activation in response to light stimulation of YFP- and Chr2-expressing axons in hippocampal slices, 4 weeks after the injection of a Cre-dependent Chr2 with or without Cre-dependent P301L mutant tau expression in wild-type or Wfs1-Cre mouse brains. (D) Right: Quantification of CA1 neuronal activity after light activation of YFP-expressing channelrhodopsin axons in hippocampal slices during the total 20 s of recording after light stimulation [one-way ANOVA, tau effect *F*_(2,18)_ = 8.30 **P < 0.01; Tukey’s post hoc, *P < 0.05 and **P < 0.01. Wt-Chr2 group: *n* = 8 animals; Wfs1-Chr2 group: *n* = 7 animals; Wfs-Chr2-tau group: *n* = 6 animals]. (**E**) Quantification of CA1 neuronal activity after light activation of YFP and Chr2-expressing axons in hippocampal slices during only the first 2 s after light stimulation [one-way ANOVA, tau effect *F*_(2,18)_ = 7.19 **P < 0.01; Tukey’s post hoc, *P < 0.05 and **P < 0.01. Wt-Chr2 group: *n* = 8 animals; Wfs1-Chr2 group: *n* = 7 animals; Wfs-Chr2-tau group: *n* = 6 animals]. (**F** to **H**) Shown is a representative parasagittal section of Wfs1-Cre mouse brain injected unilaterally with AAV.PHP.eB-Flex-hM3D-mCherry (DREADDs activator) into the hippocampal CA1 region and AAV2/6-Flex-P301L mutant tau or AAV2/6-Flex-GFP injected unilaterally into the MEC. Four weeks after viral vector injection and 90 min after intraperitoneal injection of clozapine at 0.3 mg/kg, hippocampal CA1 sections were immunostained with (F) anti-mCherry antibody (red) and anti-GFP antibody (green); (G) anti-human tau HT7 antibody and (H) anti–c-fos antibody. Scale bars, 200 μm. (**I**) Quantification of c-fos staining in (H) for the contralateral (internal control for clozapine) and ipsilateral sides [two-way ANOVA, interaction effect *F*_(1,22)_ = 19.86; Tukey’s post hoc, ****P < 0.0001; ns, not significant; control: *n* = 6 animals; P301L mutant tau: *n* = 7 animals].

We next used a combined approach to characterize the functional consequences of tau propagation to the CA1 brain region of Wfs1-Cre mice. We first assessed the excitability of CA1 by optogenetic stimulation of ECII Wfs1^+^ axonal terminals after the stereotactic injection of Cre-inducible AAV expressing humanized channelrhodopsin-2 (hChR2) carrying the H134R mutation fused to enhanced yellow fluorescent protein (EYFP; AAV2/9-DIO-hChR2-EYFP with or without injection of Cre-inducible AAV2/6-Flex-P301L mutant tau) into the MEC region of Wfs1-Cre mouse brain (fig. S17A). At 4 weeks after injection, we confirmed the expression of hChR2-EYFP in CA1 stratum lacunosum of Wfs1-Cre mice (wild-type mice injected with AAV2/9-DIO-hChR2-EYFP were the negative control) in sagittal hippocampal sections placed onto a multielectrode array (Fig. 4A). We found a near-complete absence of neuronal activity under baseline conditions (Fig. 4B) and strong CA1 neuronal activity in response to electrical stimulation of the dentate gyrus (Fig. 4C), indicating functional dentate gyrus-CA3-CA1 connectivity under all conditions. Optical stimulation of hChR2-EYFP Wfs1^+^ axons present in CA1 stratum lacunosum elicited induction of neuronal firing in CA1 (Fig. 4D), without affecting dentate gyrus neuronal activity (fig. S17, B and C). CA1 neuronal firing by optical stimulation was reduced by coexpression of human P301L mutant tau in ECII Wfs1^+^ neurons (Fig. 4, D and E). Thus, ECII Wfs1^+^ neurons established an excitatory connection to CA1, which was impaired by human P301L mutant tau expression. Next, we assessed the excitability of CA1 pyramidal neurons using a chemogenetic approach with Designer Receptor Exclusively Activated by Designer Drugs (DREADDs). We injected Cre-inducible DREADDs activator (AAV.PHPeB-Flex-hM3D-mCherry) into CA1 and AAV2/6-Flex-GFP with or without AAV2/6-Flex-P301L mutant tau into the MEC of Wfs1-Cre mice (fig. S18A). At 4 weeks after injection, we confirmed the presence of a GFP^+^ axonal bundle projecting from ECII Wfs1^+^ neurons to CA1 stratum lacunosum and mCherry expression in CA1 pyramidal cells indicating expression of human muscarinic acetylcholine receptor 3 DREADD (hM3D) (Fig. 4F). We confirmed tau propagation in the CA1 of mice injected with the AAV2/6-Flex-P301L mutant tau (P301L mutant tau group) as expected (Fig. 4G). Clozapine was used to trigger activation of DREADDs, and activation of CA1 neurons was monitored by induction of the immediate early gene c-fos 90 min after the injection of clozapine (Fig. 4H) (14). There was almost no c-fos signal in the contralateral CA1 of both GFP control and human P301L mutant tau groups (Fig. 4I). Strong induction of c-fos expression was observed on the ipsilateral side of the clozapine-injected mouse brains, which was reduced in the P301L tau group (Fig. 4, H and I). These data suggest that ECII-derived tau propagation to CA1 pyramidal neurons suppressed intracellular signaling in these cells after chemogenetic activation.

### Neurophysiological and cognitive dysfunction in ECII-CA1 tau mice

**Fig. 5.**
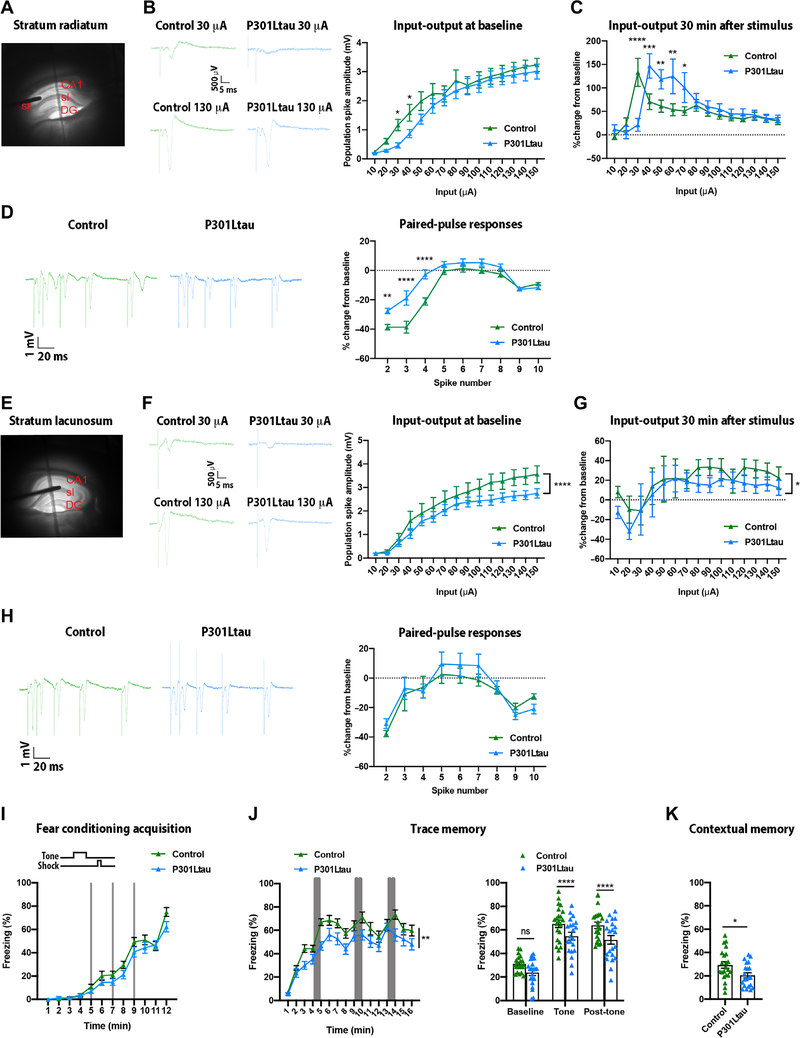
Effects of tau propagation in mouse hippocampal CA1 on neuronal excitability and behavior. (**A**) Photomicrograph of a hippocampal slice from a Wfs1-Cre mouse brain showing location of the stimulating electrode in the stratum radiatum (sr) and recording electrode in the stratum pyramidale (CA1). (**B**) Left: Shown are representative field potential responses of hippocampal CA1 slices to 30 and 130 μA of stimuli in the stratum radiatum for control and P301L mutant tau mouse brains. (B) Right: Shown are mean input (tetanic stimulus) and output (response) plots for hippocampal slices of wild-type control mice and P301L mutant tau mice [two-way ANOVA, tau effect *F*_(1,292)_ = 9.80, ***P < 0.001, Fisher’s least significance difference post hoc test *P < 0.05, control: *n* = 12 animals; P301L mutant tau: *n* = 10 animals]. (**C**) Mean change in population spike amplitude in response to a tetanic stimulus in hippocampal slices of wild-type control and P301L mutant tau mice 30 min after stimulation [two-way ANOVA, interaction effect *F*_(14,295)_ = 4.07, ****P < 0.0001; Fisher’s least significance difference post hoc test, *P < 0.05, **P < 0.01, ***P < 0.001, and ****P < 0.0001; control: *n* = 12 animals; P301L mutant tau: *n* = 10 animals]. (**D**) Left: Representative paired pulse responses for hippocampal slices of wild-type control mice compared to P301L mutant tau mice. (D) Right: Shown is a plot of percent change in responses compared to the first stimulus of a series of nine interspike intervals (10 stimuli in total) for hippocampal slices of wild-type control mice compared to P301L mutant tau mice [two-way ANOVA, interaction effect *F*_(8,180)_ = 5.25 ****P < 0.0001; Fisher’s least significance difference post hoc test, **P < 0.01 and ****P < 0.0001; control: *n* = 12 animals; P301L mutant tau: *n* = 10 animals]. (**E**) Photomicrograph of a hippocampal slice showing the location of the stimulating electrode in the stratum lacunosum and the recording electrode in the CA1. (**F**) Left: Shown are representative field potential responses of hippocampal CA1 slices of wild-type control and P301L tau mouse brains to 30 and 130 μA of stimuli of the stratum lacunosum. (F) Right: Shown is the mean input (tetanic stimulus) and output (response) plots for wild-type control compared to P301L mutant tau mice [two-way ANOVA, tau effect *F*_(1,293)_ = 19.43, ****P < 0.0001; control: *n* = 12 animals; P301L mutant tau mice: *n* = 10 animals]. (**G**) Shown is the mean change in population spike amplitude in response to a tetanic stimulus in wild-type control compared to P301L mutant tau mice 30 min after the stimulus [two-way ANOVA, tau effect *F*_(1,266)_ = 4.49, *P < 0.05; control: *n* = 12 animals; P301L mutant tau mice, *n* = 10 animals]. (**H**) Left: Shown are representative paired pulse responses in hippocampal slices from wild-type control and P301L mutant tau mice. (H) Right: Shown is a plot of percent change in responses in hippocampal slices compared to the first stimulus of a series of nine interspike intervals (10 stimuli total) for wild-type control and P301L mutant tau mice [two-way ANOVA, tau effect *F*_(1,197)_ = 0.90, not significant, Fisher’s least significance difference post hoc test, *n* = 11 to 14 slices per group]. (**I**) Shown is a time course of freezing in the fear conditioning test during training on day 1 observed in control mice and in Wfs1-Cre mice after injection of an AAV vector carrying P301L human mutant tau into the MEC [two-way ANOVA on repeated measures, tau effect *F*_(1,41)_ = 3.56, not significant; control: *n* = 22 animals; P301L mutant tau: *n* = 21 animals]. (**J**) Shown is trace memory on day 2 of the fear conditioning test [left: two-way ANOVA on repeated measures, tau effect *F*_(1,41)_ = 8.89, **P < 0.01; right: two-way ANOVA, tau effect *F*_(1,123)_ = 18.24, ****P < 0.0001, Sidak post hoc test, control: *n* = 22 animals; P301L mutant tau mice: *n* = 21 animals]. (**K**) Shown is testing of contextual memory on day 3 of the fear conditioning test (unpaired *t* test, *t* = 2.491, *P < 0.05; control: *n* = 22 animals; P301L mutant tau mice: *n* = 21 animals). See table S2 for sample details for (C) to (E) and (G) to (L).

To ascertain whether the trans-synaptic spread of tau into CA1 affected the excitability of CA1 pyramidal neurons, we bilaterally injected AAV2/6-Flex-P301L mutant tau or AAV2/6-Flex-tdTomato into MEC of Wfs1-Cre mouse brain (fig. S18B). We performed extracellular field potential recordings in the stratum pyramidale while stimulating afferent fibers in either the stratum radiatum (Fig. 5A) or stratum lacunosum of acute hippocampal slices 4 weeks after injection. Mean input-output curves were generated by application of a series of increasing input stimuli to the stratum radiatum that elicited increasing field synaptic responses and population spike amplitudes (Fig. 5B). For the stratum radiatum to CA1 stratum pyramidale pathway, the input-output curve was right-shifted in the P301L mutant tau group at stimulus intensities between 10 and 40 μA (Fig. 5B), indicating reduced excitability at low stimulus amplitudes in the P301L mutant tau group compared to the tdTomato control group. After a 30-min baseline, a high-frequency theta burst stimulus was applied to the stratum radiatum to evoke long-term potentiation. Thirty minutes after the tetanic stimulus was applied, both control and P301L mutant tau groups demonstrated strong (>100%) long-term potentiation that did not differ in terms of maximum degree of potentiation (Fig. 5C). Consistent with the input-output relationship, the maximal degree of potentiation in hippocampal slices from mice in the P301L mutant tau group was seen at a slightly higher stimulus input compared to control (40 versus 30 μA, respectively). Field potential responses to paired stimuli were examined at multiple interpulse intervals to determine whether the nature of paired pulse responses differed between the two groups (Fig. 5D). We observed a reduced paired pulse inhibition at pulse intervals of <50 ms in the P301L mutant tau group compared to the control group, whereas the paired pulse responses to longer interspike intervals did not differ between the groups. An identical series of field potential recordings were performed while stimulating the stratum lacunosum in different hippocampal slices prepared from the same animals (Fig. 5, E to G). Distinct from the input-output responses seen on stimulation of stratum radiatum, stimulation of stratum lacunosum resulted in a rightward shift in responses to higher amplitude stimuli ranging from 1.1 to 1.5 mA in the P301L mutant tau group compared to the control group, whereas responses to the lower stimulus intensities did not differ (Fig. 5F). Thus, the P301L mutant tau group reached asymptote at 90 μA, whereas the control group did not reach asymptote even at 150 μA, indicating the possibility of reduced achievable excitability of CA1 neurons receiving stratum lacunosum input in the P301L mutant tau group. Furthermore, we observed a small difference in the degree of potentiation of the population spike after a high-frequency tetanizing stimulus (Fig. 5G), but no difference in the paired pulse responses to stimulation of the stratum lacunosum between the two groups (Fig. 5H). Overall, these ex vivo experiments in Wfs1-Cre mouse brain hippocampal slices indicated a dysfunctional temporoammonic pathway and altered excitability of CA1 neurons in the P301L mutant tau group compared to the control group.

Last, we examined whether tau spread from ECII to CA1 could affect episodic memory in a task consisting of associating objects in time and space, which relies on the EC-hippocampus network in animals and humans (15, 16). MEC is engaged in general information about space and interacts with the hippocampus in this function (17). In addition, the temporoammonic pathway between EC and CA1 is involved in acquisition and reinstitution of trace memories when associated stimuli are presented with a delay to mice (8). Trace memories, defined as the temporal association of discontinued events, are driven by direct inputs from ECIII neurons projecting to CA1 pyramidal neurons (18) and modulated by ECII Wfs1^+^ neurons projecting to the stratum lacunosum interneurons through feedforward inhibition (8). CA1 pyramidal neurons are also directly involved in the formation and retrieval of episodic memories, including contextual memories (19–21). Thus, we used the trace fear conditioning task to test temporal association and contextual memories in Wfs1-Cre mice injected bilaterally with either the AAV2/6-Flex-P301L mutant tau or AAV2/6-Flex-tdTomato as a control. Wfs1-Cre mice in both control and P301L mutant tau groups behaved normally during the acquisition phase of the fear conditioning task (Fig. 5I). However, the P301L mutant tau group showed impaired trace fear memory, as evidenced by an overall reduction in their freezing time and by a reduced freezing time during the tone presentations and during the post-tone times (Fig. 5J). In addition, the P301L mutant tau group showed a deficit in contextual fear memory, as indicated by their reduced freezing time compared to the control group (Fig. 5K). Thus, ECII-CA1 tau mice displayed a deficit in both trace and contextual memories, reflecting dysfunction in the CA1 and the temporoammonic pathway.

## DISCUSSION

Here, we show robust transfer of human tau from ECII to CA1 pyramidal neurons and direct tau transfer between axon terminals of ECII Wfs1^+^ neurons and dendrites of CA1 pyramidal neurons in ECII-CA1 tau mice, resulting in decreased excitability of CA1 pyramidal neurons and impaired trace and contextual memory formation. Our ECII-CA1 tau mouse model may recapitulate tau pathology progression and suggests that neurophysiological dysfunction of CA1 pyramidal neurons may underlie hippocampal learning impairments documented in the early stages of AD.

We also observed tau propagation in cortex and thalamic regions beyond CA1. This was consistent with the known projections of ECII Wfs1^+^ neurons to CA1, subiculum, parasubiculum, contralateral entorhinal cortex, and CA1 (8) and the projections of ECII to thalamic nuclei (22). This is relevant to AD pathology as neurofibrillary tangles first appear in the transentorhinal cortex before spreading to the anterior hippocampus, first in the CA1 and then in the subiculum and parasubiculum, followed by ECIII and dentate gyrus (1, 23, 24). Tau then propagates to the adjacent limbic and temporal cortex, association isocortex, and even further to primary sensory cortex (1, 23, 24). Last, thalamic nuclei are also known to present tau pathology in AD (25, 26). Whereas there is still a debate regarding the possible differential hemispheric lateralization origin of tau accumulation, it progressively affects both brain hemispheres in patients with AD.

Expression of AAV2/6-Flex-P301L mutant tau in ECII-Wfs1^+^ cells of Wfs1-Cre mice provided evidence of tau propagation specifically to CA1 pyramidal neurons. The stereotactic coordinates for entorhinal cortical injection into Wfs1-Cre mice were key as injection of AAV2/6-Flex-P301L mutant tau at dorsoventral 3.7 instead of 3.3 resulted in spread of HT7^+^ tau in the dentate gyrus and CA1. This suggested that not all ECII Wfs1^+^ neurons project only to CA1 through the temporoammonic pathway. Some ECII Wfs1^+^ neurons proximal to the lateral entorhinal cortex (i.e., the ventral side) may be a combination of reelin^+^ stellate and calbindin^+^ pyramidal neurons (27) and therefore could also project to the dentate gyrus through the perforant pathway. The regional heterogeneity of Wfs1^+^ neurons in MEC requires further investigation to understand their precise identity and projection to hippocampal neurons.

The rodent MEC may correspond to the dorsocaudal part of the entorhinal cortex in primates, as both are close to the presubiculum and parasubiculum (7, 28). Calbindin^+^ pyramidal cells are organized in a hexagonal grid of patches in ECII of murid rodents, some bats, and humans and are known to participate in theta oscillations similar to reelin^+^ cells (7, 29, 30). Thus, impairment of ECII Wfs1^+^ neurons by tau accumulation may affect grid cell function in the entorhinal cortex as previously reported in stellate neurons of a tau transgenic mouse model (31). Our study along with several other studies reports Wfs1^+^ pyramidal cells in the human brain ECII. Most of the human studies investigating ECII have used antibodies against calbindin to detect pyramidal neurons (4, 6). Whether Wfs1^+^ neurons represent all of the calbindin^+^ neuronal population remains elusive. Our study identified p-tau accumulation in Wfs1^+^ pyramidal neurons in the early Braak stages of neurodegeneration and also suggests that Wfs1^+^ cells may be vulnerable to p-tau accumulation as indicated by the steep decline of PHF1^+^Wfs1^+^ cells in the postmortem brain samples of AD patients with CDR score 3. Further study is necessary for thorough quantitative analysis of the distribution of Wfs1^+^ and p-tau^+^Wfs1^+^ neurons in the various stages of AD.

Kitamura *et al.* (8) have shown that Wfs1^+^ ECII axons connect to CA1 indirectly through interneurons in CA1 stratum lacunosum, although minor populations project directly to distal dendrites of CA1 neurons. We observed direct contact of ECII Wfs1^+^ axonal fibers to dendrites and dendritic spines of CA1 pyramidal cells and stratum lacunosum interneurons by both high-resolution confocal microscopic imaging and immunoelectron microscopy. This finding was further validated using rabies virus–mediated monosynaptic tracing. We also confirmed the direct synaptic contacts of ECII Wfs1^+^ neurons with the CA1 and observed excitation of CA1 pyramidal cells by optogenetic stimulation of ECII Wfs1^+^ axonal terminals, which was reduced by the accumulation of tau in ECII Wfs1^+^ cells. This indicated an alteration of the excitation pathway from entorhinal cortex Wfs1^+^ neurons to the CA1 after tau propagation. AT8^+^ varicose-like dendritic damage was observed temporally in CA1 stratum lacunosum in postmortem brains from patients with AD in Braak stages II and III (32), suggesting perturbed connections in the CA1 stratum lacunosum during tau pathology development. Our finding was further validated by field recording results showing a reduction in CA1 pyramidal neuron excitability after the repetitive electrical stimulation of stratum lacunosum. Schaffer collateral connectivity was spared from tau-induced synaptic damage, which was confirmed by both multielectrode array and field recordings, and correlated with lack of tau distribution in the dentate gyrus of ECII-CA1 tau mice. Accumulation of tau in mouse CA1 pyramidal cells ultimately impaired memory forming intracellular signaling in these cells, as determined by suppressed CA1 c-fos expression after chemogenetic activation of the CA1 pyramidal cells with tau accumulation.

Trace fear memory is regulated by ECIII input and through feedforward inhibition from ECII Wfs1^+^ cells (8). We expected that tau accumulation in ECII Wfs^+^ neurons would limit the feedforward inhibition, leading to CA1 hyperexcitability and stronger associative memory formation. However, we observed a reduced CA1 excitability associated with reduced trace fear memory formation in the ECII-CA1 tau mice. This could be due to tau accumulation in CA1 neurons and in their specific afferents from ECII, but with CA3 afferents remaining unaffected. In addition, ECII-CA1 tau mice showed impairments in contextual learning. As this is mainly regulated through the lateral entorhinal cortex-CA3-CA1 and entorhinal cortex-amygdala axes (33), impaired memory storage in CA1 cells may be responsible for the impairment in contextual learning in ECII-CA1 tau mice, which is in line with reduced c-fos induction after chemogenetic excitation of CA1 neurons.

We analyzed the data separately by sex and notably did not find sex differences between mice at this age, and so data from both sexes were combined. The incidence of AD and other dementias is higher in women (34). Several explanations have been proposed, including women’s higher survival at older ages and men’s higher mortality due to cardiovascular diseases, yet these explanations are not conclusive (34). Further investigations are needed to explore sex differences in ECII-CA1 tau propagation in humans.

There are several limitations to our study. The ECII-CA1 AAV tau mouse model needs further improvement to avoid viral contamination of Wfs1^+^ CA1 pyramidal neurons, which we observed in about 6% of HT7^+^ neurons in the mouse CA1. The alternative approach using transgenic mice would not be suitable because transgenic mice expressing Cre-inducible human tau crossed with Wfs1-Cre mice would express the transgene in CA1 Wfs1^+^ neurons and Wfs1^+^ ECII neurons, making it difficult to study tau propagation. We are confident, however, that the tau propagation observed in this mouse model was through the transport of human tau from Wfs1^+^ ECII pyramidal neurons to hippocampal regions as injection of AAV2/6-Flex-P301L mutant tau at dorsoventral 3.7 induced tau propagation to the dentate gyrus where Wfs1^+^ cells were not detected. The infected donor cells and recipient cells alternatively can be distinguished by using an AAV vector coexpressing tau and a reporter gene, which does not have cytotoxicity. Another limitation of our approach is due to the short incubation time used after AAV injection. It normally takes 6 months or longer to develop fibrillar tau accumulation in conventional P301L mutant tau transgenic mice (35, 36). Wfs1^+^ cells are vulnerable to tau accumulation after viral injection, making it unsustainable to express P301L mutant tau for a period of time sufficient for fibrillar tau to accumulate. This problem could be alleviated by using drug-inducible tau transgenic mice. The third limitation of our ECII-CA1 tau mouse model was that the P301L mutation in tau did not induce tau pathology seen in AD cases but was necessary to induce robust tau aggregation in a short time period. Thus, propagation of human tau after injection of human tau seeds, such as AD brain–derived extracellular vesicles, into mouse brain should be investigated in future studies (37). Last, the number of human postmortem brain samples was limited, and so further study with a larger number of brain samples will be needed to validate our findings.

Our ECII-CA1 tau mouse model may recapitulate some aspects of tau pathology seen in postmortem brains from patients with AD in early Braak stages and with low cognitive dementia rating scores. Deficits in our ECII-CA1 tau mice included neurophysiological dysfunction of CA1 pyramidal neurons and associative learning impairments, which could explain some of the deficits observed clinically in patients with AD. The temporoammonic pathway we have studied here requires further evaluation to comprehensively understand how direct and indirect ECII-CA1 pathways contribute to mediating tau propagation and impaired hippocampal learning.

## MATERIALS AND METHODS

### Study design

The objective of this study was to determine the role of mouse Wfs1^+^ pyramidal neurons in the MECII-CA1 pathway during the development of early tau pathology in mice. We generated ECII-CA1 tau mice with early AD tau pathology by combining a Wfs1-Cre transgenic mouse model with surgical injection of an AAV vector carrying human P301L mutant tau into mouse brain. This ECII-CA1 tau mouse model was made by the stereotactic injection of Cre-inducible AAV2/6 expressing P301L mutant tau or tdTomato (as a negative control) into Wfs1-Cre mouse brain, which expresses Cre-inducible genes in Wfs1^+^ pyramidal neurons. The ECII-CA1 tau mouse brains were subjected to immunohistochemistry and confocal and immunoelectron microscopic imaging. Hippocampal CA1 slices were subjected to ex vivo electrophysiological field recording with a multielectrode array to determine excitability of CA1 hippocampal neurons. ECII-CA1 tau mice were subjected to behavioral testing using a fear conditioning test. Equal numbers of male and female mice were randomly assigned to each experiment. Animal numbers for assessing tau transmission were based on our previous publications (12, 38). Removal of outliers was conducted following parameters described in the statistical part of Materials and Methods. All data analyses were performed blinded to the genotype or treatment of the animals. Each animal represents an individual biological data point. Replication and sample sizes for all experiments are detailed in the figure legends.

### Animals

Male and female Wfs1-Cre mice (obtained from the Tonegawa laboratory, The Picower Institute for Learning and Memory, Massachusetts Institute of Technology) and C57BL6/J mice (Jackson Laboratory) were kept on a 12-hour light/12-hour dark schedule with access to chow ad libitum. Pregnant mothers were single-housed. Offspring were weaned at P21 and group-housed with two to five same-sex littermates per cage. The number, sex, and age of animals for each experiment are listed in each figure legend or table S2. No statistical methods were used to predetermine sample size, and randomization of samples was done according to age and litter. All animal procedures followed the guidelines of the National Institutes of Health Guide for the Care and Use of Laboratory Animals and were approved by the Boston University Institutional Animal Care and Use Committee.

### Stereotactic surgery

Stereotaxic viral and dye injections were all performed in accordance with Boston University Institutional Animal Care and Use guidelines. Mice were anesthetized using 1 to 2% isoflurane in a 95% O_2_ and 5% CO_2_ mixture. Viruses or dyes were injected by using a computer-controlled stereotaxic frame (Neurostar, Germany) equipped with a glass micropipette attached to a 10-μl Hamilton microsyringe through a microelectrode holder (World Precision Instrument, MPH6S10) filled with mineral oil. A microsyringe pump and its controller were used to control the speed of injection. The needle was slowly lowered (0.1 mm/min) to the target site, injections were done at a rate of 0.1 μl/min, and the needle remained at the target site for 10 min after the injection.

For histology, unilateral viral delivery into the right MEC of Wfs1-Cre mice was placed at Bregma coordinates: anteroposterior (AP) −4.85, mediolateral (ML) +3.45, dorsoventral (DV) −3.30. The 24- to 32-week-old Wfs1-Cre mice were injected with 700 nl of AAV2/6-Flex-P301L tau [3.34 × 10^11^ genome copy number (GC)/ml] (39) or 300 nl of AAV2/6-Flex-tdTomato (9.74 × 10^10^ GC/ml; SignaGen) or AAV2/6-Flex-GFP (3.2 × 10^12^ GC/ml; North Carolina Vector Core), and mice were subsequently perfused after the incubation periods indicated in the data for each experiment.

For comparison between depth of injection, unilateral viral delivery into the right MEC of Wfs1-Cre mice was placed at Bregma coordinates: AP −4.85, ML +3.45, DV −3.30 or DV −3.70. For monosynaptic tracing experiment, unilateral viral delivery in the right CA1 of Wfs1-Cre was placed at Bregma coordinates: AP −2.1, ML −1.7, DV −1.4. The 24- to 32-week-old Wfs1-Cre mice were injected with 300 nl of AAV2/9-Flex-EnVa-GFP [1.24 × 10^12^ GC/ml (40), The Viral Vector Core, Salk Institute], followed 2 weeks later by an injection at the same coordinates of 300 nl of AAV2/9-Flex-g-deleted-rabies-mCherry [3.62 × 10^9^ transduction unit (TU)/ml, The Viral Vector Core, Salk Institute]. Animals were euthanized and brains were collected 7 days after injection.

For DREADD experiment, unilateral viral delivery into the right CA1 of Wfs1-Cre was placed at Bregma coordinates: AP −2.1, ML −1.7, and DV −1.4. The 24- to 32-week-old Wfs1-Cre mice were injected with 300 nl of AAV-PHP.eB-DIO-hM3D-mcherry (5.36 × 10^9^ GC/ml; Addgene 44361-PHPeB) and 700 nl of AAV2/6-FlexP301L tau. Four weeks after injection, clozapine (0.3 mg/kg; Hello Bio, HB6149) was intraperitoneally injected and animals were euthanized for brain collection 90 min after injection.

For optogenetic stimulation, unilateral viral delivery into the right MEC of Wfs1-Cre was placed at Bregma coordinates: AP −4.85, ML +3.45, and DV −3.30. The 24- to 32-week-old Wfs1-Cre mice were injected with 300 nl of AAV2/9-EF1a-DIO-hChR2(H134R)-EYFP-WPRE-HGHpA (1.08 × 10^−11^ GC/ml; Addgene 20298) and 700 nl of AAV2/6-Flex-P301L tau.

For electrophysiological field recording and behavior, bilateral viral delivery into the MEC of Wfs1-Cre was placed at Bregma coordinates: AP −4.85, ML +3.45, DV −3.30 and AP −4.85, ML −3.45, DV −3.30. The 24- to 32-week-old Wfs1-Cre mice were injected with 700 nl of AAV2/6-Flex-P301L tau or 300 nl of AAV2/6-Flex-tdTomato, and animals were tested at 4 weeks after injection.

### Immunohistochemistry of human postmortem brain tissue

Postmortem brain tissue was obtained from human subjects with cognitive status assessed by CDR scores and Mini-Mental State Examination, with neuropathology classified by Braak stages for tau and Thal stages for amyloid β (41) and with short postmortem intervals, as indicated in table S1. The specimens were provided by C. Bouras from the Department of Psychiatry, University of Geneva School of Medicine, Geneva, Switzerland (42). The materials were fixed in 4% paraformaldehyde (PFA). Tissue sections encompassing the entorhinal cortex and hippocampus were cut at 50 μm using a vibratome and stored at 4°C in phosphate-buffered saline (PBS) with 0.1% sodium azide until stained. Free-floating sections were washed in tris-buffered saline (TBS; pH 7.0), and antigen retrieval was performed by heating in tris-EDTA [10 mM tris and 1 mM EDTA (pH 9.0)] for 30 min at 70°C and cooling down in the same buffer to room temperature for another 30 min. The sections were then washed three times for 10 min each in TBS and subject to blocking by incubation in a solution of 5% normal goat serum, 5% bovine serum albumin (BSA), and 0.3% Triton X-100 in TBS at room temperature for 1 hour. The sections were incubated for 64 hours at 4°C with primary antibodies raised against WFS1 (1:1000; rabbit polyclonal, #26995–1-AP, Proteintech, Rosemont, IL) and against p-tau, either PHF1 or CP13 (1:1000; mouse monoclonal provided by P. Davies, Feinstein Institute for Medical Research, Northwell Health, Manhasset, NY, USA) diluted in antibody diluent solution (5% BSA and 0.1% Triton X-100 in TBS). Rabbit immunoglobulin G (IgG) or mouse IgG1 isotype controls (#3900 and #5415, respectively, Cell Signaling Technology, Danvers, MA) were used as the negative controls. After three 10-min washes in TBS, the sections were incubated for 2 hours at room temperature with a goat anti-mouse secondary antibody conjugated to Alexa Fluor 594 (5 μg/ml; #A11005, Invitrogen, Carlsbad, CA) and biotinylated goat anti-rabbit secondary antibody (5 μg/ml; #BA-1000, Vector Laboratories, Burlingame, CA). The sections were washed thrice in TBS and incubated with anti-mouse secondary antibody conjugated to Alexa Fluor 594 (5 μg/ml; #A11005, Invitrogen) and streptavidin–Alexa Fluor 488 complex (1:500; #S11223, Invitrogen) for 2 hours at room temperature. The extended incubation with primary and secondary anti-rabbit antibodies allowed for complete penetration of the sections by PHF1 and CP13. After three washes in TBS, the sections were mounted on glass slides and dried at 50°C for 1 hour. Slides were then washed in TBS, and lipofuscin autofluorescence was quenched by treatment with TrueBlack (Biotium, Fremont, CA), washed thrice, counterstained with DAPI (250 ng/ml; Sigma-Aldrich), and coverslipped under VECTASHIELD antifade mounting medium (Vector Laboratories).

### Immunohistochemistry of mouse brain tissues

Brains were collected after perfusion and fixation with 4% PFA in PBS, followed by overnight postfixation and cryoprotection in 30% sucrose and embedding in an OCT compound (Sakura). Brains were frozen on dry ice and sectioned by cryostat at 30 μm into sagittal or coronal sections.

For propagation studies, sections collected at every 150-μm interval were used for immunohistochemistry. Sections were subjected to antigen retrieval with pH 9.0, 10 mM tris base/1 mM EDTA/PBS at 95°C for 20 min followed by 20 min at room temperature in the tris-EDTA working solution. The sections were blocked in 5% normal goat serum/5% BSA/0.3% Triton-X in PBS. Sections were incubated with the primary antibody diluted with 5% BSA/0.1% Triton-X in PBS for 24 hours at 4°C. Primary antibodies are as follows: WFS1 (1:1000; Proteintech, 26995–1-AP); HT7 (1:1000; Invitrogen, MN1000); Tau13 (1:500; BioLegend, 835201); red fluorescent protein (RFP; 1:1000; Rockland Immunochemicals, 200–101-379); phospho-tau(Ser^202^,Thr^205^) AT8 (1:300; Invitrogen, MN1020); misfolded tau MC1 (1:300) and CP13 (1:300), both gifts from P. Davies; anti-GFP (1:100; Santa Cruz Biotechnology, sc-9996); GAD67 biotin–conjugated (1:250 Millipore, MAB5406B); anti-beta subunit Cholera Toxin (CTB) antibody (1:500 Abcam, ab62429); c-fos (1:5000 Synaptic Systems, 226003); Reelin (1:100; Invitrogen, PA5–47537); mCherry (1:5000; Biorbyt, orb153320). Sections were then washed three times in PBS and incubated with Alexa Fluor 488–, Alexa Fluor 594–, Alexa Fluor 568–, Alexa Fluor 647–conjugated antibodies or streptavidin–Alexa Fluor 488–conjugated antibody (all from Invitrogen; 1:1000) for 2 hours at room temperature.

### Imaging studies

Fluorescence images of the human brain sections were acquired using a CLSM 780 confocal microscope (Carl Zeiss Microscopy, Jena, Germany), using a 20×/0.8 DICII and a 63×/1.4 Oil DICIII objective and DPSS 561–10, 405 diode and Argon lasers at excitation wavelengths of 405, 488, and 561 nm. Confocal stacks in layers II and III of the entorhinal cortex were imaged at 512 × 512 pixel resolution with a Z-step of 1 μm (for 20×) or 0.31 μm (for 63×), a pinhole setting of 1 Airy Unit for the red wavelength and optimal settings for gain and offset. Images are presented as maximum intensity projections of the Z-stack, adjusted for brightness and contrast. For quantification of neurons containing Wfs1 and PHF1, nine confocal z-stack images (at 20×) evenly distributed across ECII from each case was analyzed (Fig. 1). Neurons with Wfs1, PHF1, and Wfs1/PHF1 colocalization in the entire field were manually counted by an investigator blind to the subjects’ information, using the pointer tool in FIJI/ImageJ (43). The total number of DAPI^+^ cells was automatically counted using Imaris X64 9.5.1. Images were converted to a Native Imaris File format, followed by spot analysis with an estimated diameter of 4 μm. The total number of spots was used as the estimated number of DAPI^+^ cells in the Z-stack. The percentage of Wfs1^+^ cells in ECII was calculated by dividing the average number of Wfs1^+^ cells by the average number of DAPI^+^ cells. The percentage of Wfs1^+^PHF1^+^cells was calculated by dividing the number of Wfs1^+^PHF1^+^ cells by the total number of Wfs1^+^ cells.

Large field fluorescence images of the mouse brain sections were acquired using a epifluorescence microscope (Nikon Eclipse Ti, Japan), using Plan Fluor ELWD 20× Ph1 DM objective at a 1024 × 1024 pixel resolution at excitation wavelengths of 405, 488, 561, and 647 nm. Confocal microscopy images were obtained using 20×/0.75 HC Plan-Apochromat CS2 and 63× oil immersion/1.4 HC Plan-Apochromat CS2 objective (Leica TCS SP8 Lightning, Germany) and diode laser at excitation wavelengths of 405, 488, 552, and 647 nm at 1024 × 1024 pixel resolution with a system optimized z-step and a pinhole setting of 1 Airy Unit for all wavelengths and optimal settings for gain and offset.

For immunohistochemistry (IHC) analysis of HT7^+^ and c-fos^+^ neurons, we used FIJI software. For HT7 IHC, we analyzed CA1 and dentate gyrus in brain sections of 30 μm every 100 μm from bregma 3.2 to 0.9 mm. We first took large images of each brain section and used DAPI and WFS1 signal to identify the exact localization of the section compared to Allen Atlas for bregma coordinate (44). CA1 and dentate gyrus were then identified on the basis of DAPI staining, clearly allowing us to distinguish CA1 from CA2 and subiculum and to identify dentate gyrus. CA1 and dentate gyrus were manually cropped and areas were measured on each image. To determine HT7^+^ neurons in CA1 and dentate gyrus areas, we quantified the total number of HT7^+^ neurons in CA1 and dentate gyrus of each image to be able to measure the propagation in these brain regions. To do so, we manually defined for each image the value of intensity needed to count a neuron as positive. This value was at least two times higher than the background signal measured on a DAPI^+^ and HT7^−^ neuron of the corresponding image. We counted the total number of HT7^+^ neurons for CA1 and dentate gyrus of each image and then calculated the density of HT7^+^ neurons for each image by dividing the total number of HT7^+^ neurons in CA1 and dentate gyrus by CA1 and dentate gyrus areas. Final results were presented for each animal quantified (*N* = 7 total) from each brain section (Fig. 1D). In addition, we calculated the sum of the density of HT7^+^ neurons per animal from bregma 3.2 to 0.9 mm and presented it separately (Fig. 1D, top right). For c-fos IHC, we followed similar method except that we focused on the CA1 region. Final results were presented as average density per animal (*N* = 6 to 7 total) (Fig. 4I). For high-resolution imaging of GFP^+^ dendrites and tau^+^ axons, confocal image stacks were acquired using a Leica SPE or SP8 confocal microscope equipped with a 40×/1.3 or 63× 1/4 oil immersion HC Plan-A pochromat objectives (Leica Lightning, Germany) and 488, 542, and 647 diode lasers, at a voxel resolution of 0.1 × 0.1 × 0.3 μm. Image stacks were then deconvolved using AutoQuant (RRID:SCR_002465; Media Cybernetics, Bethesda, MD).

Colocalization analyses of tau and GFP were conducted as described (45). For each image stack, ImageJ was used to first threshold (Otsu) each color channel of GFP (channel 1, c1) and tau (channel 2, c2) signal. To assess colocalization between tau and GFP, an ImageJ colocalization plug-in was first used to identify regions of interest (ROIs) of colocalized pixels (above signal threshold for each channel) and create a binary mask across the confocal stack. The Z-maximum projections of individual channels and the colocalized binary mask ROI stacks were obtained, and particle analyses were done to calculate percent area labeled in each individual channel and the colocalized points using ImageJ (RRID:SCR_003070) (46), as described (45). These values for percent labeled area in each channel and percent colocalized area were used to calculate Manders’ colocalization coefficients (M1 = fraction of c1 colocalized with c2; M2 = fraction of c2 colocalized with c1) for each channel as follows: M1, fraction of GFP (c1) colocalized with tau (c2) = percent area colocalized/total percent area GFP label; M2, fraction of tau (c2) colocalized with GFP (c1) = percent area colocalized/total percent area tau label. For 3D construction of the images, Imaris X64 9.5.1 was used. Images were converted to Native Imaris File format, and snapshots of the rotated 3D construction were taken. For the videos, an animation of the 3D construction was created using a 360° horizontal rotation, and the video was captured as 1600 × 1200 ultra extended graphics array (4:3) at 25 frames/s.

### mRNA in situ hybridization

In vitro transcription of complementary RNA (cRNA) probes against the AAV 3′UTR transgene WPRE was constructed in the pBluescript II KS(−) vector, and cRNA probe against human P301L tau under the T7 promoter was synthesized as double-stranded DNA fragment as previously described (12). The 645–base pair reverse complement of the 3′UTR posttranslational regulatory element WPRE sequence in the AAV vector was used for the cRNA probe of AAV transgenes. Digoxigenin (DIG)–labeled cRNA riboprobe was synthesized using DIG labeling mix (catalog no. 1 277 073, Roche Diagnostics GmbH, Mannheim, Germany) and Riboprobe In Vitro Transcription Systems (catalog no. P1420, Promega, Madison, WI) and purified using ProbeQuant G-50 microcolumns (catalog no. GE28–9034, GE Healthcare, Boston, MA). Enhanced fluorescence in situ hybridization was conducted for the detection of human tau and WPRE mRNA on brain tissue sections using anti-digoxigenin antibody conjugated with horse-radish peroxidase (anti–DIG-POD) Fab fragments (catalog no. 11 207 733 910, Roche, Branford, CT), with the TSA Plus Cy5 fluorescence system (catalog no. NEL745, PerkinElmer, Waltham, MA). After in situ hybridization, the tissue sections were processed for immunofluorescence as described above to detect WFS1 or HT7.

### Electron microscopy

Brains were collected after perfusion and fixation with 4% PFA and 0.2% glutaraldehyde in PBS followed by overnight postfixation and cryoprotection in 30% sucrose. Brains were then sectioned in the sagittal plane at 100 μm on a Leica VT1000S Vibratome and stored in antifreeze solution [30% ethylene glycol, 30% glycerol, 40% 0.05 M phosphate buffer (PB)] at −20°C. Sections corresponding to the dorsal hippocampus were first processed for fluorescence labeling of GFP, together with either tau or tdTomato (as described above). Matched tissue sections adjacent to tissue sections with robust fluorescence labeling were then selected for dual pre-embedding immunoelectron microscopy processing for verification of synaptic connections, according to (47, 48). Tissue was first incubated in 50 mM glycine for 1 hour at room temperature and then rinsed (three times for 10 min in 0.01 M PBS) and incubated for 2 hours in preblock solution (0.01 M PBS, 5% BSA, 5% normal donkey serum, and 0.025% Triton-X). Sections were then coincubated overnight at 4°C in primary antibodies as follows: one set was incubated in anti-GFP (Novus Biologicals, NB600–308; 1:1000; rabbit) and anti–p-tau (HT7, Invitrogen, MN1000; 1:1000, mouse) and the other in anti–enhanced GFP (Novus Biologicals, NB600–308; 1:1000, rabbit) and anti-RFP (cross-reacting to tdTomato, Rockland, 200–101-379; 1:500, mouse), diluted in incubation buffer [0.1 M PB with 0.2% BSA-c (acetylated BSA, Aurion) 1% normal donkey serum (NDS) and 0.025% Triton-X]. Tissue was rinsed (three times for 10 min in 0.01 M PBS) and then coincubated in the following secondary antibodies: biotinylated donkey anti-rabbit IgG (1:200; Jackson ImmunoResearch) and 0.8 nm gold-conjugated donkey anti-mouse IgG (1:100; Aurion) diluted in incubation buffer containing 0.1% gelatin. For each primary and secondary antibody incubation period, tissue was incubated twice in a low-wattage Pelco Biowave Pro (Ted Pella) for 10 min at 150 W (at 25°C) to improve penetration. Tissue was then washed in 0.1 M PB with 0.2% BSA-c, followed by an incubation in 20 mM sodium citrate (pH 7.1; 5 min, 4°C) and rinsed 1 to 2 min in distilled water (dH_2_O). Sections were incubated in silver enhancement solution (Aurion) for 60 to 90 min at room temperature. After rinses in 20 mM sodium citrate (pH 7.1) for 5 min, dH_2_O, and 0.1 M PB (pH 7.4, three times for 10 min), tissue was incubated in avidin-biotin horseradish peroxidase (HRP) enzyme complex solution (Vector PK6100 ABC elite kit, Vector Laboratories) first in the microwave (two times for 3 min at 150 W) and then at room temperature for 5 to 10 min. Tissue was then washed three times for 10 min in 0.1 M PB and stained using 3,3′-diaminobenzidine (DAB) substrate (Vector Laboratories) for 2 min. Sections were washed (three times for 10 min in 0.1 M PB) and postfixed in a 6% glutaraldehyde, 2% PFA solution using the microwave (at 150 W) until sample temperature was >30°C. Sections were incubated overnight in 6% glutaraldehyde and 2% PFA solution at 4°C, as previously described (47, 48).

After immunohistochemistry and postfixation, tissue was rinsed in 0.1 M PB (three times over 20 min, 25°C) for processing and embedding for EM, as previously described (47, 48). Sections were incubated in two-stage osmium using the microwave: first in 1% osmium with 1.5% KFe in 0.1 M PB, followed by 1% osmium in 0.1 M PB (each step 4 min in Biowave at 100 W, 10 min on shaker at 25°C). After rinsing (three times for 10 min in 0.1 M PB and two times in dH_2_O), tissue was dehydrated in increasing gradients of alcohol (50 to 100% ethanol), with an en block 1% uranyl acetate counterstaining step in 70% ethanol for 30 min on a tissue shaker (25°C). Tissue was infiltrated with Araldite resin (Ted Pella) using propylene oxide (15 min in 100%, followed by a 1:1 propylene oxide:Araldite resin for 3 hours; 100% Araldite overnight in a vacuum dessicator). Tissue was then flat-embedded in araldite resin between aclar plastic (Ted Pella) and cured at 60°C for 48 hours. The whole stratum lacunosum-moleculare with tdTomato- or tau-labeled fibers and GFP^+^ dendrites was microdissected (average block face, ~400 μm × 200 μm) and re-embedded in Araldite blocks for cutting. Resin-embedded tissue blocks were cut using a Leica ultramicrotome and diamond knife, first into 1-μm semi-thin sections, which were stained with toluidine blue for anatomical orientation. A short series of 5 to 10 ultrathin sections were cut at 50 nm and mounted on pioloform-coated copper grids. The entire tissue block was scanned to identify structures labeled with gold or DAB. Labeled structures were confirmed in the adjacent serial sections and photographed at ×20,000 magnification and 80 kV using a JEOL 100CX transmission electron microscope and Gatan digital camera. Synapses were identified using classic criteria based on (49). For the quantification, all tau^+^ boutons and their postsynaptic targets were classified according to the morphology (spine versus dendritic shaft) and immunogold labeling of GFP in CA1 pyramidal neuronal dendrites. These counts were pooled to determine the distribution of postsynaptic targets of tau^+^ boutons.

### Protein extraction and ELISA

Mouse brains were dissected to isolate frontal cortices (brain tissue from bregma 3.56 to 1.18 mm) frontal cortex, CA1-enriched, and EC regions, followed by flash freezing on dry ice. Protein extraction was conducted by homogenizing tissue samples in 8 volumes of 5 M guanidine hydrochloride (Sigma-Aldrich, G4505–100G) in 50 mM tris-HCl (pH 8.0) on ice. The samples were vortexed for 4 hours at room temperature and diluted 10-fold with cold PBS with 1× protease and phosphatase inhibitor cocktail (Halt/Thermo Fisher Scientific, PI78443). The samples were centrifuged at 16,000*g* for 20 min at 4°C, and the supernatant was collected to determine protein concentration by BCA assay kit (Pierce/Thermo Fisher Scientific, 23225). For the detection of total tau and p-T181 tau, the following commercial ELISA kits were used: Human tau (total) ELISA kit (Invitrogen, KHB0042) and Human tau (pT181) ELISA kit (Invitrogen, KHO0631). The ELISA method consists of capture and detection antibodies and detection using streptavidin Poly-HRP (Pierce/Thermo Fisher Scientific, 21140), 3,3′,5,5′-Tetramethylbenzidine solution (Thermo Fisher Scientific, N301), and Stop solution (Thermo Fisher Scientific, N600) with optical density at 450-nm read using a microplate reader (BioTek Instruments). The dynamic range of the detection of total tau was 31 to 2000 pg/ml, and pT181 tau was 15.6 to 1000 pg/ml according to the manufacturer’s instructions.

### Optogenetic stimulation and multielectrode array measurements

After 4 weeks after injection, mice were euthanized, brains were collected, and acute slices were generated (50). Briefly, the brain was collected after perfusion with ice-cold artificial cerebrospinal fluid (ACSF). First, the brain was cut sagitally into 100-μm sections from lateral 3.72 mm to lateral 3.0 mm for immunohistochemical analysis of the injection site. Second, sagittal sections of 300 μm were generated on a Compresstome for multielectrode array. The sections were transferred into a nest beaker containing recovery ACSF and incubated at 33°C for 30 min. Next, the nest beaker was removed from the heating bath and let to recover at room temperature for 45 min. Three hippocampal sections from each hemisphere were placed in a 24-well Lumos MicroElectrode Array plate (Axion Biosystems, M384-tMEA-24OPT), and the hippocampus was aligned with the electrodes. The sections were held down with harps, and ACSF was added to the wells. The plate was placed in the Maestro-Z at 37°C and 5% CO_2_. A baseline was established by recording with no stimulation for 20 min. The activity of the sections was recorded when they were subjected to pulses of 50% blue light intensity at 10-ms intervals for 20 min. Next, two electrodes on dentate gyrus were stimulated electrically with biphasic stimulation pulse at 0.5 V for 400 μs at 10-ms intervals for 20 min. Last, the sections were stimulated with pulses of 25% blue light intensity at 10-ms intervals for 20 min. These recordings were exported to MATLAB to obtain the number of spikes in CA1 during stimulation. The data were plotted and analyzed using Prism software.

### Electrophysiology

#### Brain slice preparation

Wfs1 mice were deeply anesthetized with isoflurane and then sacrificed by immediate decapitation. The brains were rapidly extracted and hemisected, and the right side of the brain was placed in oxygenated (95% O_2_ and 5% CO_2_) ice-cold Ringer’s solution containing 25 mM NaHCO_3_, 124 mM NaCl, 1 mM KCl, 2 mM KH_2_PO_4_, 10 mM glucose, 2.5 mM CaCl_2_, and 1.3 mM MgCl_2_ (pH 7.4; Sigma-Aldrich, St. Louis, MO). The right hemisphere was placed against a 3% agar block for support and sliced at 300-μm thickness with a Leica VT1000S vibrating brade microtome (Leica Microsystems) yielding four coronal slices containing the dorsal hippocampus. Slices were maintained at 33.3°C in oxygenated Ringer’s solution in a holding chamber for equilibration for at least 1 hour before field potential recordings. After this equilibration period, slices were transferred to submersion-type recording chambers at room temperature (Harvard Apparatus, Holliston, MA) affixed to the stages of Nikon E600 infrared–differential interference contrast microscopes (Micro Video Instruments, Avon, MA). During recordings, slices were continuously perfused with oxygenated room temperature Ringer’s solution at a rate of 2.5 ml/min.

#### Field potential recordings

In one series of experiments, concentric bipolar tungsten stimulating electrodes (FHC, USA) were positioned in the Schaffer collaterals of the stratum radiatum, whereas recording electrodes were positioned in CA1 stratum pyramidale to assess the perforant pathway–evoked field responses. In a second series of experiments, the stimulating electrodes were placed in the striatum lacunosum-moleculare, whereas recording electrodes were positioned in CA1 stratum pyramidale to investigate the direct temporoammonic pathway–evoked field responses. Extracellular field excitatory postsynaptic potentials and population spikes were recorded during a variety of stimulation protocols. The interelectrode distance was ~200 μm. Field potential recordings were made using recording pipettes fabricated from nonheparinized microhematocrit capillary tubes with resistance of 5 to 7 megohm (Thermo Fisher Scientific, Pittsburg, PA) on a Flaming and Brown horizontal pipette puller (Model P-87, Sutter Instrument, Novato, CA) and filled with Ringer’s solution. EPC-9 and EPC-10 patch-clamp amplifiers and PatchMaster software were used for data acquisition, and FitMaster analysis software was used for data analyses (HEKA Elektronik, Lambrecht, Germany). Stimuli (100 μs in duration) were applied at 0.05 Hz at amplitudes increasing in increments of 10 μA from 0 to 150 μA, to generate input (stimulus intensity)–output (response amplitude) curves for each field [Grass S88 stimulator, and Stimulus Isolation Unit, Asai *et al.* (12)]. After establishment of the input-output relationship for single pulse, a paired pulse input-output relationship paradigm was designed with varying interpulse intervals (10, 20, 30, 50, 70, 100, 200, 500, and 1000 ms). A baseline was established at the stimulus intensity that elicited a ~50% response amplitude at 0.01 Hz for a period of 15 min. Long-term potentiation was evoked using a theta burst tetanizing stimulus consisting of five 1-s pulses of 100 Hz applied at intervals of 20 s. After application of the tetanic stimulus, the baseline was run for an additional 30- to 60-min period, and the input-output relationship for single pulse and paired pulse was again assessed.

### Fear conditioning for testing trace and contextual memory formation

Trace fear conditioning experiment was performed in the animal facility during the light cycle following the previously published method that reported the involvement of the Wfs1 temporoammonic pathway in the trace fear memory paradigm with minor modification (8).

#### Trace paradigm

Wfs1-Cre mice injected bilaterally into MEC at the coordinates AP −4.85, ML +3.45, DV −3.30 and AP −4.85, ML −3.45, DV −3.30 with either AAV2/6-Flex-tdTomato or AAV2/6-Flex-P301L tau were placed 4 weeks after the injections in the context “A” and allowed to explore for 240 s, at which point a 20-s tone (85 Db, 2000 Hz) was played, followed by a 20-s trace and then a 2-s, 0.75 mA foot shock. This was repeated two more times starting at 402 and 564 s for a total time of 706 s. On day 2 (24 hours later), mice were placed in a context “B” and allowed to explore for 240 s, at which point the same tone as day 1 was played for 60 s, followed by 180 s of no-tone (post-tone period). This was repeated two more times for a total time in the context B of 960 s. On day 3 (24 hours later), mice were placed back in context A and allowed to explore for 300 s. Freezing behavior was recorded during all the time spent in either context by an experimenter blind to the genotype and treatment.

### Statistical analyses

Data are presented as means ± SEM. Unpaired *t* tests were used for comparison of immunostained neuron densities between the CA1 and dentate gyrus mouse brain regions. Multigroup data were analyzed using one-way or two-way analysis of variance (ANOVA) followed by Tukey, Fisher’s least significance difference or Sidak post hoc tests (see figure legends) and three-way ANOVA to test for a gender effect, with post hoc multiple comparisons (Prism version 8, GraphPad Software). Tau propagation (Fig. 2D and fig. S12, A and B) and fear conditioning datasets (Fig. 5, I to K) were analyzed using two-way ANOVA on repeated measures. Normality of data distribution was assessed using Kolmogorov-Smirnov test and confirmed for every dataset except for the multielectrode array baseline data. The statistical significance was set at α = 0.05. Outliers were removed using the Robust Regression and Outlier Removal (ROUT) test, with a false discovery rate of 1%, in Prism 8.0. The R program was used to generate the heatmap. For multielectrode array experiments, we only removed outlier data when mouse brain slices were deformed at the time of the experiment (1 wild-type mouse, 1 Wfs1-Cre control mouse) or in the absence of YFP expression at the site of channelrhodopsin virus injection (1 Wfs1-Cre P301L mutant tau mouse). For the electrophysiological experiments, animals were removed on the basis of the ROUT test only (1 control mouse for stratum radiatum baseline; 1 control and 1 P301L mutant tau mouse for stratum radiatum 30 min after tetanic stimulation). For immunohistochemical and ELISA experiments, no outliers were removed. For chemogenetics experiments, one animal was removed because of the lack of mCherry signal in the CA1, indicative of no hM3D-mCherry expression. For the behavioral experiments, one animal from the P301L mutant tau group showing generalized freezing behavior (100% freezing) and two control mice presenting very low freezing behavior during the training were removed by the ROUT test.

## Supplementary Material

Supplementary Material

Video S1

Video S2

## Data Availability

All data associated with this study are present in the paper or the Supplementary Materials. A material transfer agreement (MTA) between Massachusetts Institute of Technology and Boston University School of Medicine exists for Wfs1-Cre mice produced by S. Tonegawa’s laboratory. An MTA between Albert Einstein College of Medicine and Boston University School of Medicine exists for CP13, MC1, and PHF1 mouse monoclonal antibodies produced by P. Davies’ laboratory. An MTA exists between Stanford University and Boston University School of Medicine for the AAV2/9-EF1a-DIO-hChR2(H134R)-EYFP-WPRE-HGHpA construct, originally produced by K. Deisseroth’s laboratory. MTAs between Addgene and Boston University School of Medicine exist for the AAV-PHP.eB-DIO-hM3D-mCherry construct, originally produced by the B. Roth laboratory. An MTA between the Salk Institute and Boston University School of Medicine exists for AAV2/9-Flex-EnVa-GFP and AAV2/9-Flex-g-deleted-rabies-mCherry produced by the Viral Vector Core. The AAV-Flex-P301L mutant tau vector is available through an MTA upon request to T.I.
