# Supplementary material for: Wolframin-1–expressing neurons in the entorhinal cortex propagate tau to CA1 neurons and impair hippocampal memory in mice
Source: Sci Transl Med. Author manuscript; Available in PMC 2022 Jan 17. (PMC8763211; doi:10.1126/scitranslmed.abe8455)
Supplement: Supplementary Material [file NIHMS1769104-supplement-Supplementary_Material.pdf]

Science Translational Medicine
AAAS

Supplementary Materials for

Wolframin-1–expressing neurons in the entorhinal cortex propagate tau to CA1 neurons and impair hippocampal memory in mice

Jean-Christophe Delpech *et al.*

Corresponding author: Tsuneya Ikezu, ikezu.tsuneya@mayo.edu

Sci. Transl. Med. **13**, eabe8455 (2021)
DOI: 10.1126/scitranslmed.abe8455

The PDF file includes:

Tables S1 and S2
Figs. S1 to S18
Legends for movies S1 and S2

Other Supplementary Material for this manuscript includes the following:

Movies S1 and S2
Data file S1

Case ID	Age	Sex	CDR	MMSE	Thal (Ab)	Braak (tau)	PMI (h)	Diagnosis	Cause of death
1	87	F	0	27	0	II	8	Neurologically normal, cognitively intact	carcinoma, bronchopneumonia
2	74	M	0	N/A	1	I	5	Neurologically normal, cognitively intact	myocardial infarct
3	85	M	0	29	1	I	7	Neurologically normal, no cognitive alterations	pulmonary cancer (no metastases to brain)
4	82	M	0.5	26	1	I	7	No neurologic alterations, mild cognitive impairment	undetermined
5	85	M	0.5	28	3	III	4	Fluctuating neurological status at end of life, with mild cognitive impairment, possible early stage AD	cardiorespiratory insufficiency
6	61	F	0.5	26	1	I	7	Mild cognitive impairment, possible early stage AD	carcinoma, bronchopneumonia
7	88	F	2	15	2	III	6	Cognitive decline	bronchopneumonia
8	88	M	2	13	2	III	8	Dementia, cognitive impairment	pyelonephritis
9	90	M	2	12	3	VI	2	Definite AD, severe cognitive impairment	bronchopneumonia
10	83	F	3	12	3	V	5	Definite AD, severe cognitive impairment	bronchopneumonia
11	85	M	3	11	4	V	11	Definite AD, severe cognitive impairment	bronchopneumonia
12	95	F	3	10	4	VI	4	Definite AD, severe cognitive impairment	cardiac failure

Table S1. Human subject demographics. CDR: clinical dementia rating, MMSE: mini-mental state examination, PMI: postmortem interval

Figure	Group	Male	Female	Total	Age
1-B	CDR0	2	1	3	61-95 years old
	CDR0.5	2	1	3	
	CDR 2	2	1	3	
	CDR3	1	2	3	
2-D	P301Ltau	4	3	7	5-8 months
2-D insert	P301Ltau	4	3	7	
2-F	Control	3	3	6	5-6 months
	P301Ltau	3	3	6	
3-E	P301Ltau	4	1	5	5 months
3-G	P301Ltau	2	1	3	5 months
4-B-C-D-E	WT-Chr2	4	4	8	5-8 months
	Wfs1-Chr2	4	3	7	
	Wfs1-Chr2-tau	3	3	6	
4-I	Control	3	3	6	6-9 months
	P301Ltau	3	4	7	
5-B-C-D-F-G-H	Control	7	5	12	5-6 months
	P301Ltau	5	5	10	
5-I-J-K	Control	11	11	22	5-6 months
	P301Ltau	10	11	21	
S9	P301Ltau	4	3	7	5-8 months
S11	Control	3	3	6	5-6 months
	P301Ltau	3	3	6	5-6 months
S12	DV3.3	4	3	7	5-8 months
	DV3.7	4	4	8	6-9 months
S17	WT-Chr2	4	4	8	5-8 months
	Wfs1-Chr2	4	3	7	
	Wfs1-Chr2-tau	3	3	6	

Table S2. Dataset information

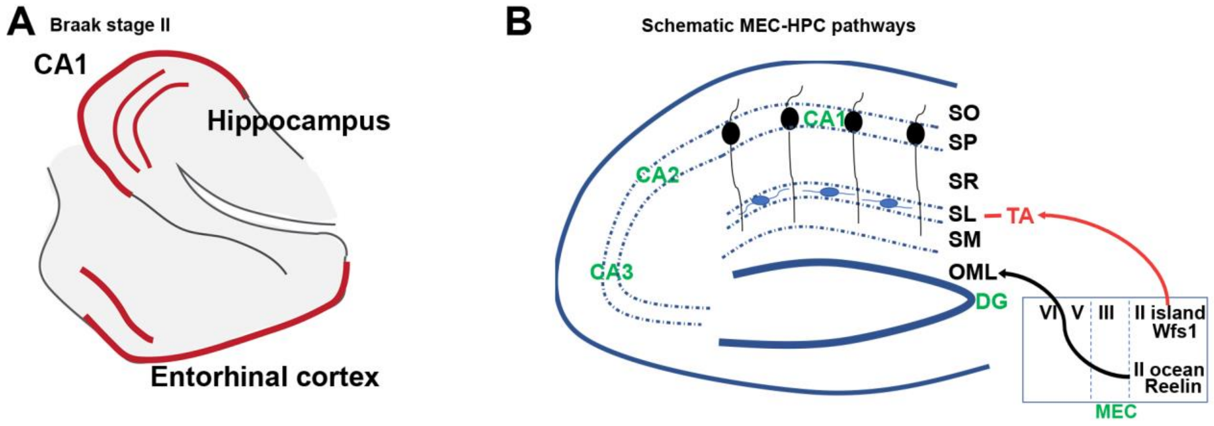

Fig. S1. **Schematic representations of early Braak stage tau positivity and entorhinal cortex - hippocampal pathway.**

(A) Human Braak stage II AT8 positivity in entorhinal cortex and hippocampus indicating AT8 positivity (red) in ECII and CA1. CA; Ammon's horn. **(B)** Schematic representation of medial entorhinal cortex (MEC) to hippocampus (HPC) temporoammonic (TA) pathway. so; stratum oriens, sp; stratum pyramidale, sr; stratum radiatum, sl; stratum lacunosum, sm; stratum moleculare, oml; outer molecular layer, CA: *Cornu Ammonis*, DG; dentate gyrus, MEC; medial entorhinal cortex.

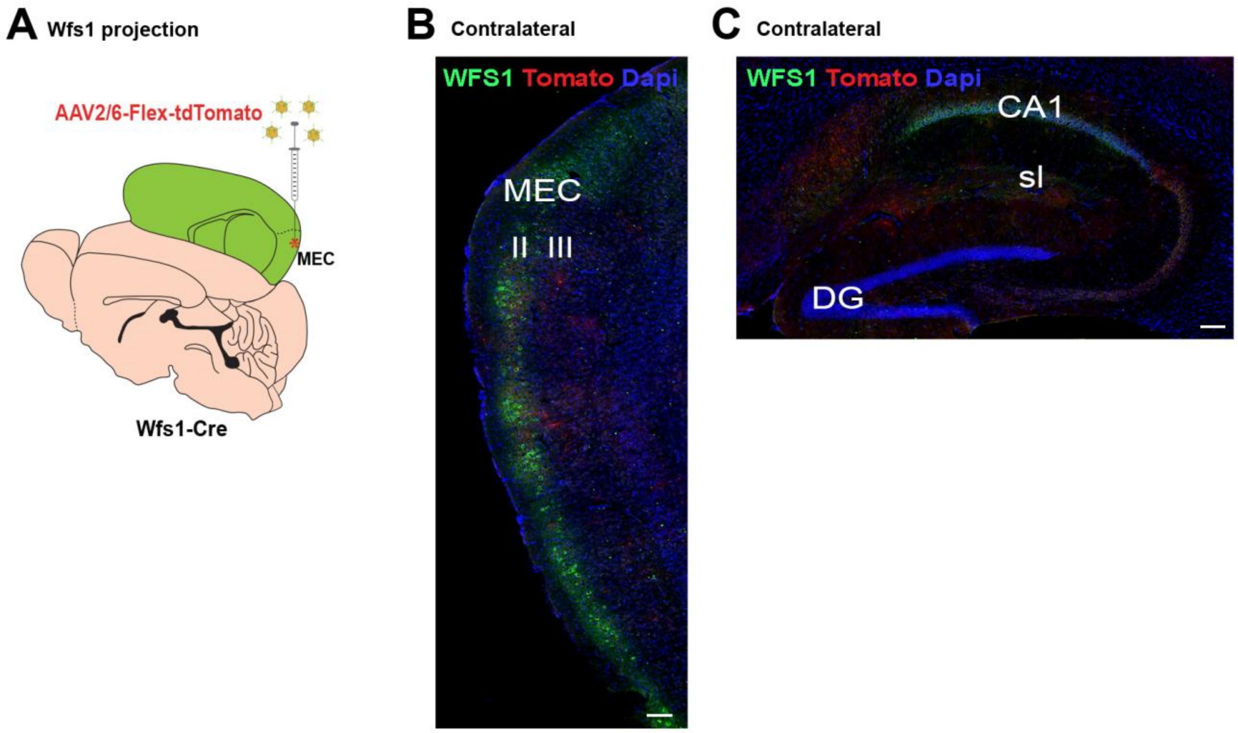

Fig. S2. **Characterization of entorhinal cortex layer II projections in contralateral side in Wfs1-Cre mice.**

(A) Diagram of the mouse brain indicating the location of AAV2/6-Flex-tdTomato unilateral injections into medial entorhinal cortex (MEC) of Wfs1-Cre mice. **(B-C)** Parasagittal section of MEC **(B)** and HPC **(C)** of Wfs1-Cre mouse injected with AAV2/6-Flex-tdTomato and immunostained with anti-Wfs1 (green) and anti-RFP (red) four weeks post injection. Scale bars, 100 µm. CA: *Cornu Ammonis*, DG; dentate gyrus, MEC; medial entorhinal cortex, sl; *stratum lacunosum*.

.

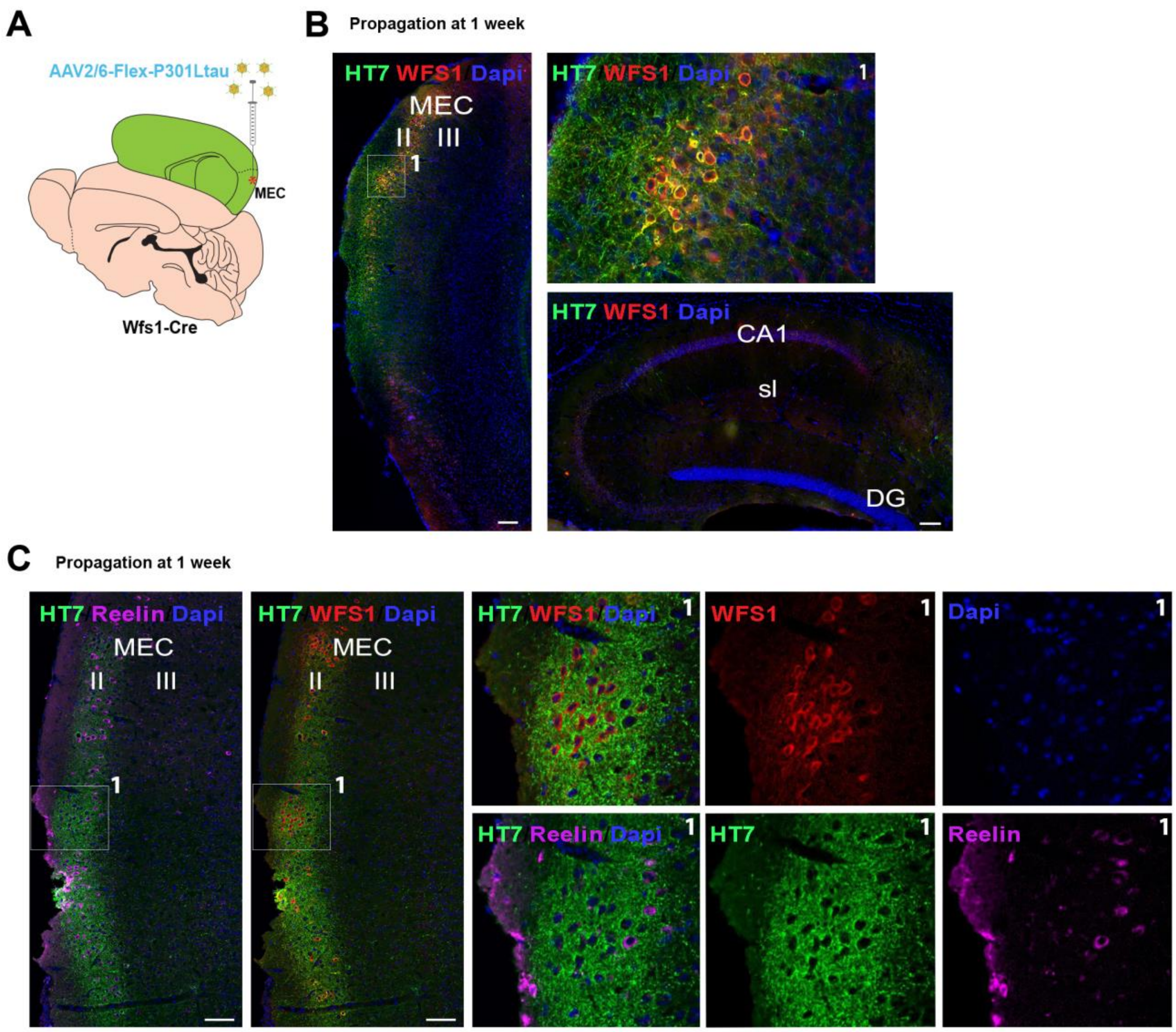

Fig. S3. **Characterization of medial entorhinal cortex to CA1 tau propagation at one week of incubation in Wfs1-Cre mice.**

(A) Diagram of the mouse brain indicating the location of AAV2/6-Flex-P301L tau unilateral injections into MEC of Wfs1-Cre mice. **(B)** Parasagittal section of MEC and HPC of Wfs1-Cre mouse injected with AAV2/6-Flex-P301L tau immunostained with anti-human tau-specific HT7 (green) and anti-Wfs1 (red) one-week post injection. **(C)** Parasagittal section of the medial entorhinal cortex (MEC) of Wfs1-Cre mouse injected with AAV2/6-Flex-P301L tau immunostained with HT7 (green), anti-Wfs1 (red) and anti-Reelin (purple) one-week post injection. Scale bars, 100 µm (n = 7 animals). CA: *Cornu Ammonis*, DG; dentate gyrus, sl; *stratum lacunosum*.

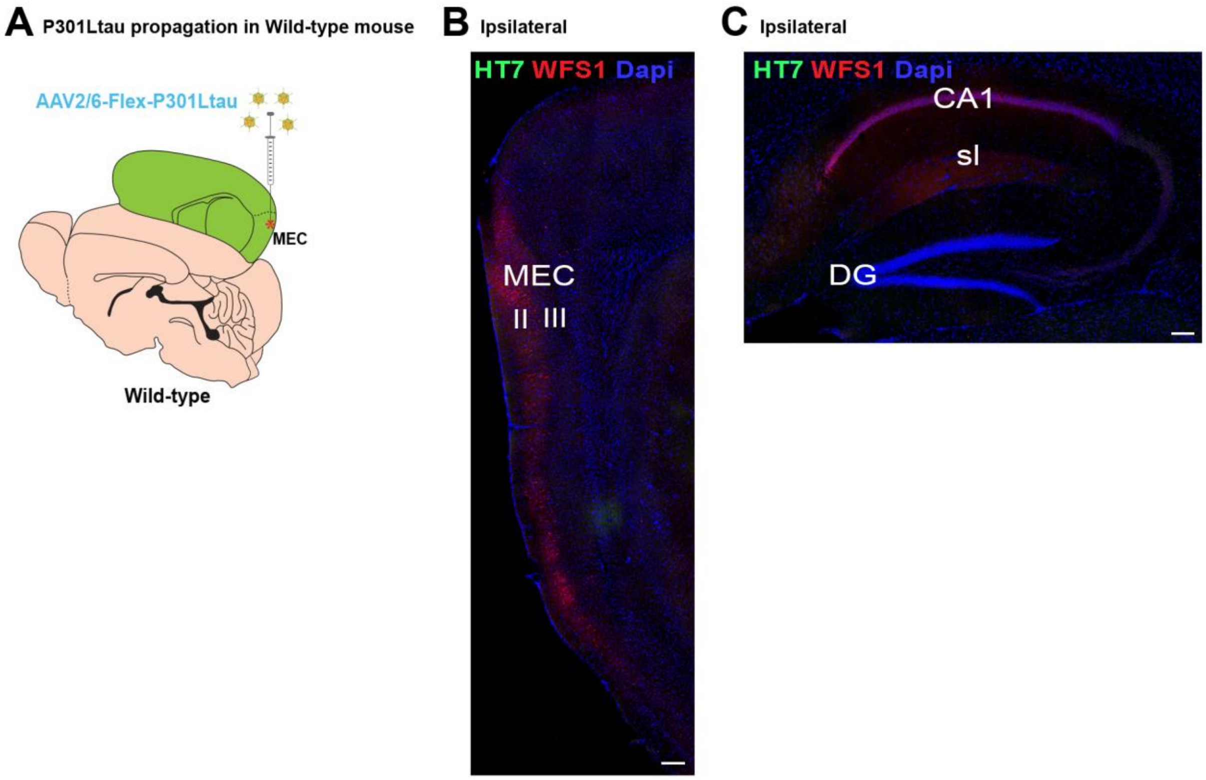

Fig. S4. Characterization of virus expression in medial entorhinal cortex and hippocampus of wild-type mice.

(A) Diagram of the mouse brain indicating location of AAV2/6-Flex-P301L tau unilateral injections into MEC of wild-type mice. **(B)** Parasagittal section of medial entorhinal cortex (MEC) of WT mouse injected with AAV2/6-Flex-P301L tau immunostained with HT7 (green), anti-Wfs1 (red) and DAPI (blue) at one-week post injection. Scale bars, 100 μm. **(C)** Parasagittal section of the hippocampus of wild-type mouse injected with AAV2/6-Flex-P301L tau and immunostained with HT7 (green), anti-Wfs1 (red) and DAPI (blue) at one-week post injection. Scale bars, 100 μm (n = 4 animals). CA: *Cornu Ammonis*, DG; dentate gyrus, sl; *stratum lacunosum*.

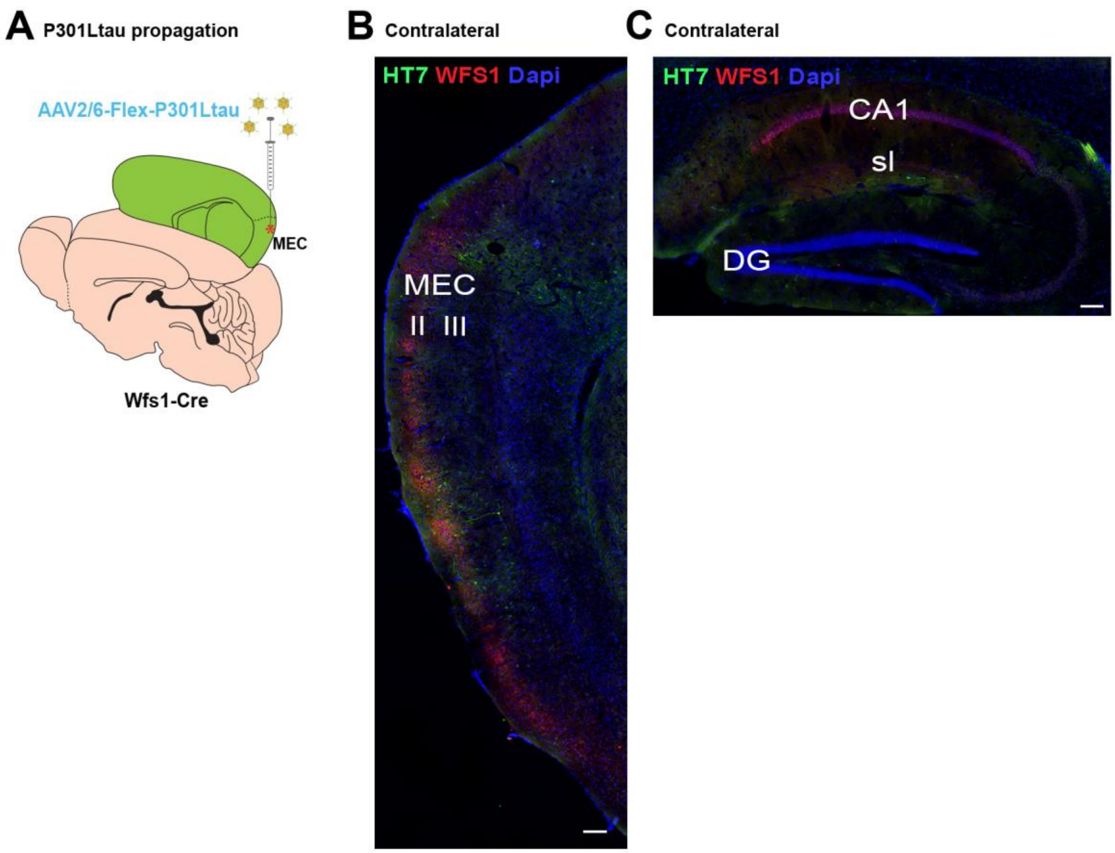

Fig. S5. Characterization of tau propagation in the contralateral side in Wfs1-Cre mice.
(A) Diagram of the mouse brain indicating the location of AAV2/6-Flex-P301L tau unilateral injection into MEC of Wfs1-Cre mice. **(B-C)** Parasagittal section of medial entorhinal cortex (MEC) **(B)** and hippocampus **(C)** of Wfs1-Cre mouse injected with AAV2/6-Flex-P301L tau and immunostained with HT7 (green), anti-Wfs1 (red) and DAPI (blue) at four weeks post injection. Scale bars = 100 µm (n = 7 animals). CA: *Cornu Ammonis*, DG; dentate gyrus, MEC; medial entorhinal cortex, sl; *stratum lacunosum*.

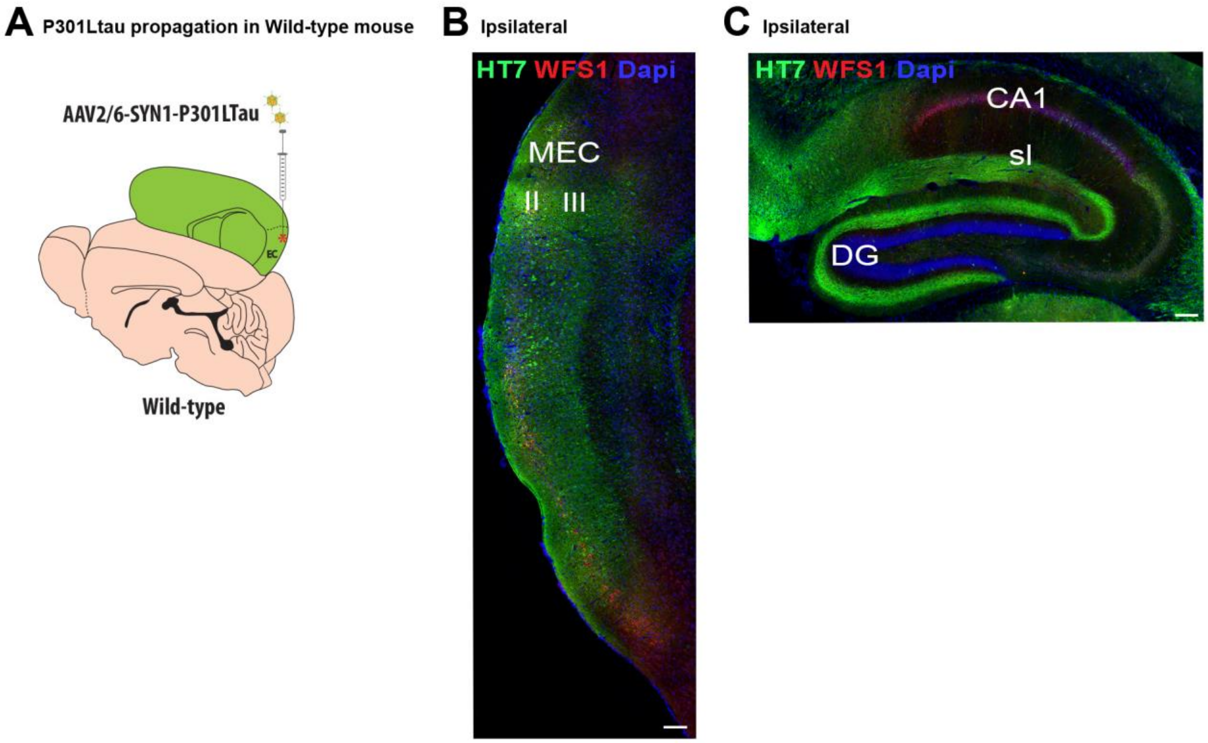

Fig. S6. Characterization of medial entorhinal cortex to CA1 tau propagation at four weeks of incubation in wild-type mice.

(A) Diagram of the mouse brain indicating the location of AAV2/6-SYN1-P301L tau unilateral injection into medial entorhinal cortex (MEC) of wild-type C57BL/6 mice. **(B-C)** Parasagittal section of MEC **(B)** and hippocampus **(C)** of wild-type mouse injected with AAV2/6-Flex-P301L tau and immunostained with HT7 (green), anti-Wfs1 (red) and DAPI (blue) at four weeks post injection. Scale bars = 100 µm (n = 3 animals). CA: *Cornu Ammonis*, DG; dentate gyrus, MEC; medial entorhinal cortex, sl; *stratum lacunosum*.

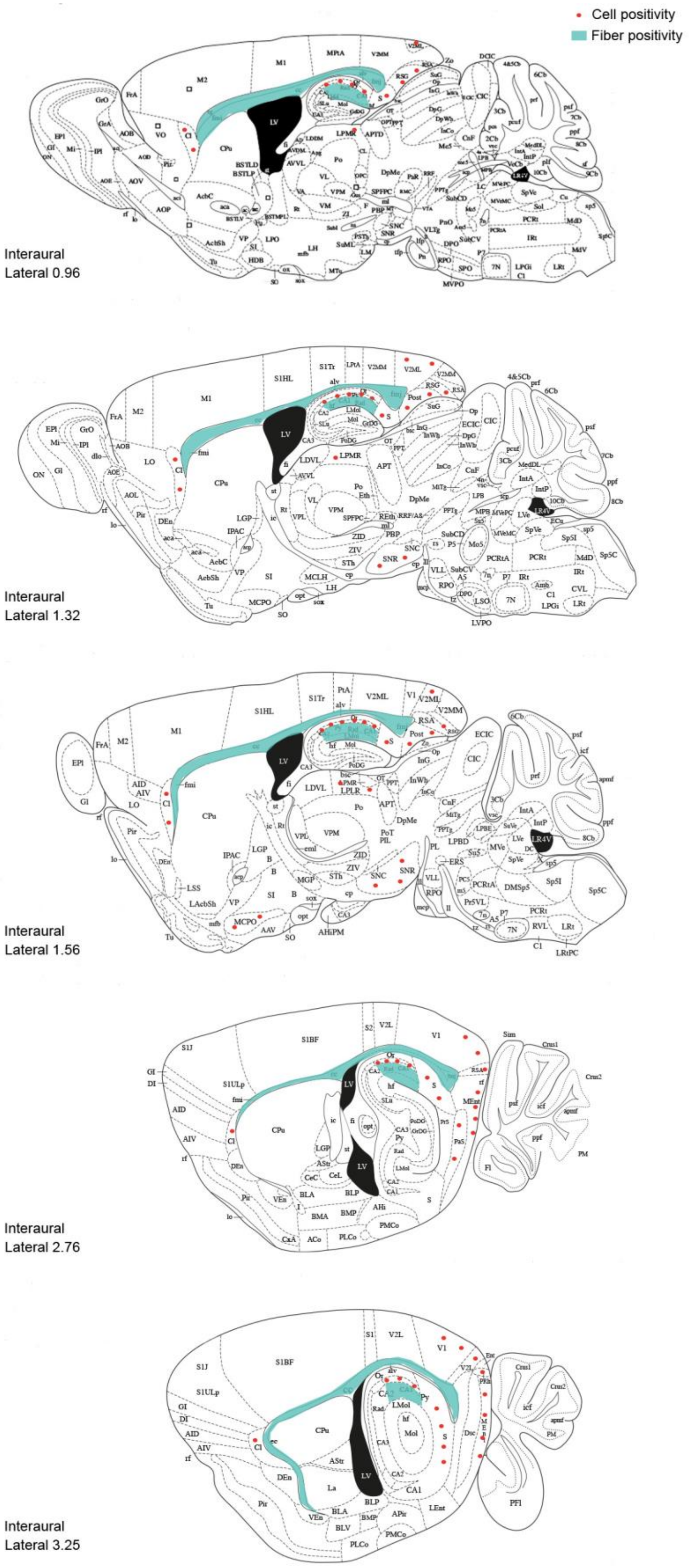

Fig. S7. Characterization of tau propagation at four weeks of incubation in Wfs1-cre mice. Schematic representation of ipsilateral parasagittal sections of the brain from interaural lateral 3.25mm to lateral 0.96 showing regions of HT7 cellular human tau positivity (red dot) in the claustrum (Cl), subiculum (S), perirhinal cortex (PRh), visual cortex (V1;V2L;V2ML), parasubiculum (PaS), retrosplenial agranular cortex (RSA), retrosplenial granular cortex (RSG), magnocellular preoptic nucleus (MCPO), rostral part of the thalamic nucleus (LPMR;LPLR), and substantia nigra pars compacta and pars reticulata (SNC;SNR), and fiber human tau positivity (blue overlay) determined by immunohistochemistry from 10× images at 4 weeks post injection of AAV2/6-Flex-P301L tau into medial entorhinal cortex of Wfs1-Cre mice.

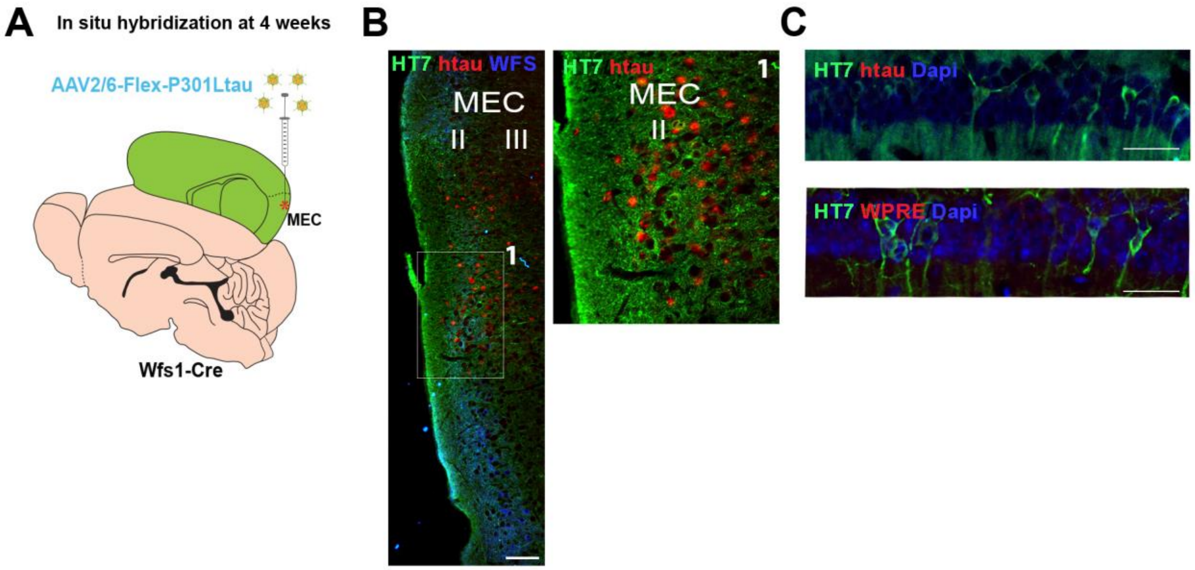

Fig. S8. Characterization of transgene RNA localization in medial entorhinal cortex and hippocampus of Wfs1-Cre mice.

(A) Diagram of the mouse brain indicating location of AAV2/6-Flex-P301L tau unilateral injections into MEC of Wfs1-Cre mice. **(B)** Parasagittal section of medial entorhinal cortex (MEC) of Wfs1-Cre mouse injected with AAV2/6-Flex-P301L tau and immunostained with HT7 (green) and DAPI (blue) and *in situ* hybridized with htau (human *MAPT* mRNA) cRNA probe (red) at four weeks post injection. Scale bars = 100 µm. **(C)** Parasagittal section of the HPC of Wfs1-Cre mouse injected with AAV2/6-Flex-P301L tau immunostained with HT7 (green) and DAPI (blue) and *in situ* hybridized with htau probe or *WPRE* cRNA probe (red) at four weeks post injection. Scale bars = 50 µm (n = 7 animals). MEC; medial entorhinal cortex, MAPT; microtubule-associated protein tau, WPRE, woodchuck hepatitis virus posttranscriptional regulatory element.

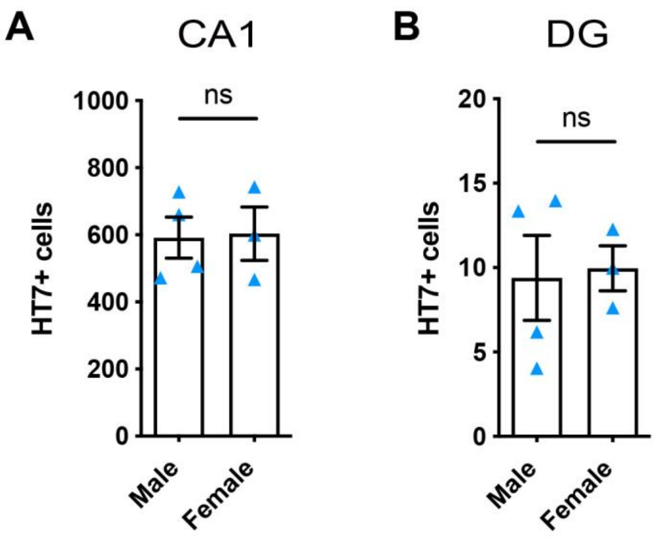

Fig. S9. Quantification of human tau propagation to hippocampus in male and female Wfs1-Cre mice.

Quantification of HT7$^+$ neurons in CA1 **(a)** and dentate gyrus (DG) **(b)** of Wfs1-Cre male and female mice injected with AAV2/6-Flex-P301L tau and immunostained with HT7 at four weeks after medial entorhinal cortex injection (Average: unpaired t-test, CA1: t=0.1206, DG: t=0.1796, ns; not significant, n = 4 Males; n = 3 females). See **Table S2** for sample details. CA: *Cornu Ammonis*, DG; dentate gyrus.

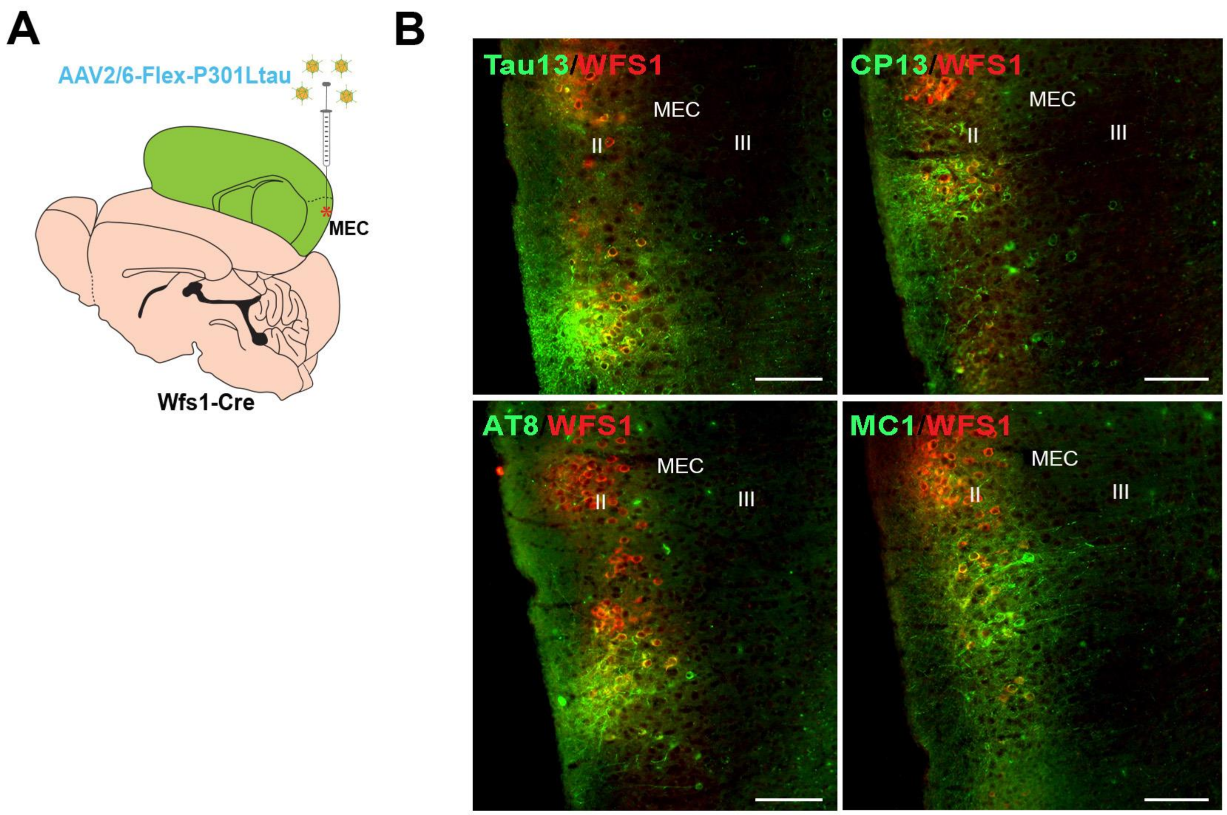

Fig. S10. Characterization of tau accumulation with multiple epitopes in the entorhinal cortex.

(A) Diagram of the mouse brain indicating the location of AAV2/6-Flex-P301L tau unilateral injection into medial entorhinal cortex (MEC) of Wfs1-Cre mice. **(B)** Parasagittal section of MEC of Wfs1-Cre mouse injected with AAV2/6-Flex-P301L tau immunostained with Tau13 (human tau), CP13 (pSer^{202} tau), AT8 (pSer^{202}/pThr^{205} tau) or MC1 (misfolded tau) (green) and anti-Wfs1 (red) at four weeks post injection. Scale bars = 100 µm (n = 4 animals). MEC, medial entorhinal cortex

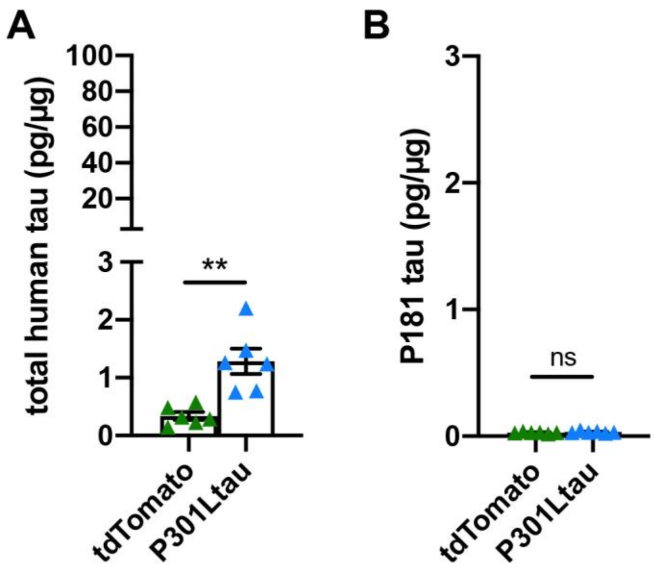

Fig. S11. Quantification of human tau in the frontal cortex.

ELISA quantification of human tau **(a)** and pT181 tau **(b)** in frontal cortices-enriched tissue of Wfs1-Cre mice injected with AAV2/6-Flex-P301L tau at four weeks post injection (Student's t-test, total tau: t=4.148, pT181 tau: t=1.191, **P<0.01, n = 6 animals per group). See **Table S2** for sample details.

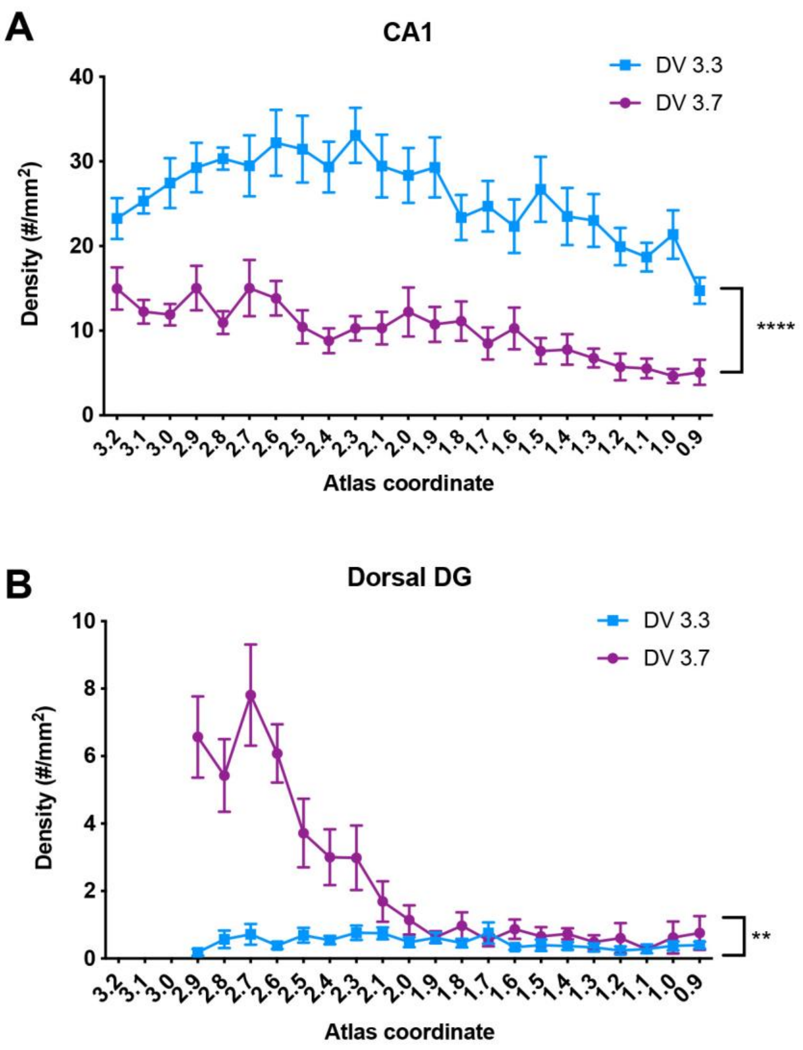

Fig. S12. Quantification of tau propagation at four weeks of incubation in Wfs1-cre mice at dorsoventral 3.3 and 3.7 medial entorhinal cortex injection depth.

(a) Quantification of $HT7^+$ neurons in CA1 of Wfs1-Cre mice injected with AAV2/6-Flex-P301L tau at dorsoventral (DV) 3.3 and 3.7 and immunostained with anti-human tau HT7 at four weeks after medial entorhinal cortex injection (two-way ANOVA on repeated measures, injection depth effect $F_{(1,13)}$=49.04 ****P<0.0001; DV3.3 n = 7 animals; DV3.7 n = 8 animals). **(b)** Quantification of $HT7^+$ neurons in dentate gyrus (DG) of Wfs1-Cre mice injected with AAV2/6-Flex-P301Ltau at DV3.3 and DV3.7 and immunostained with anti-human tau HT7 at four weeks after MEC injection (two-way ANOVA on repeated measures, injection depth effect $F_{(1,13)}$=17.38 **P<0.01; DV3.3 n = 7 animals; DV3.7 n = 8 animals). See **Table S2** for sample details. DV; dorsoventral, CA: *Cornu Ammonis*, DG; dentate gyrus.

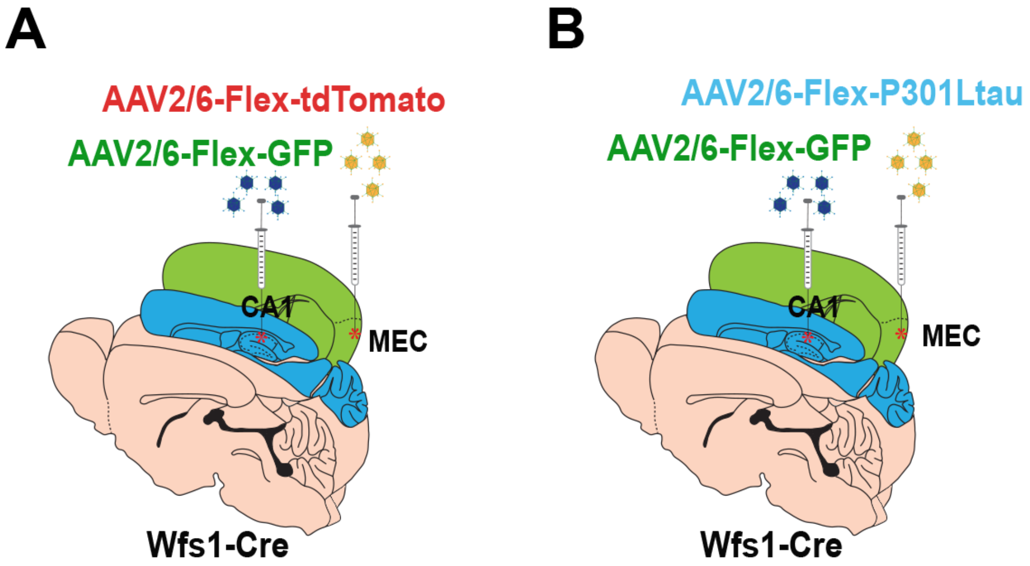

Fig. S13. Diagram of the mouse brain indicating the location of the viral injections into Wfs1-Cre mice.

(A) Diagram of the mouse brain indicating the location of AAV2/6-Flex-tdTomato unilateral injections into the medial entorhinal cortex (MEC) and AAV2/9-Flex-GFP unilateral injections into CA1 of Wfs1-Cre mice. **(B)** Diagram of the Wfs1-Cre mouse brain indicating the location of AAV2/6-Flex-P301L tau unilateral injections into the MEC and AAV2/9-Flex-GFP unilateral injections into the CA1 hippocampus. CA: *Cornu Ammonis*, MEC; medial entorhinal cortex, GFP; green fluorescent protein.

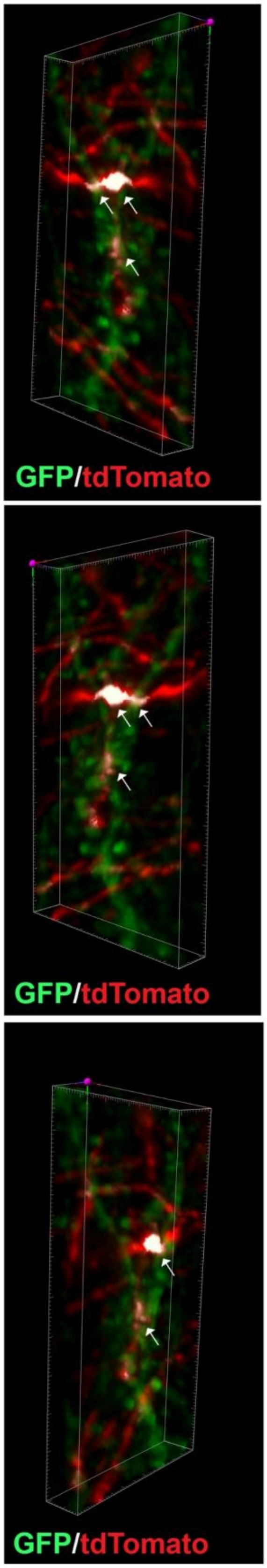

Fig. S14. 3D reconstruction of *stratum lacunosum* connections as shown in Fig. 3B.

Rotated images of the 3D reconstruction of CA1 *stratum lacunosum* region imaged by high-resolution confocal microscopy showing colocalization (white) of tdTomato (red) and GFP (green) signal taken from a parasagittal section of HPC of Wfs1-Cre mice injected with AAV2/6-Flex-tdTomato (red) unilaterally into medial entorhinal cortex and AAV2/9-Flex-GFP (green) unilaterally into CA1 and immunostained by anti-mCherry/anti-GFP antibodies. Scale bars (red/green arrows at the corner) = 20 µm. See also **Video S1**. CA; *Cornu Ammonis*, GFP; green fluorescent protein

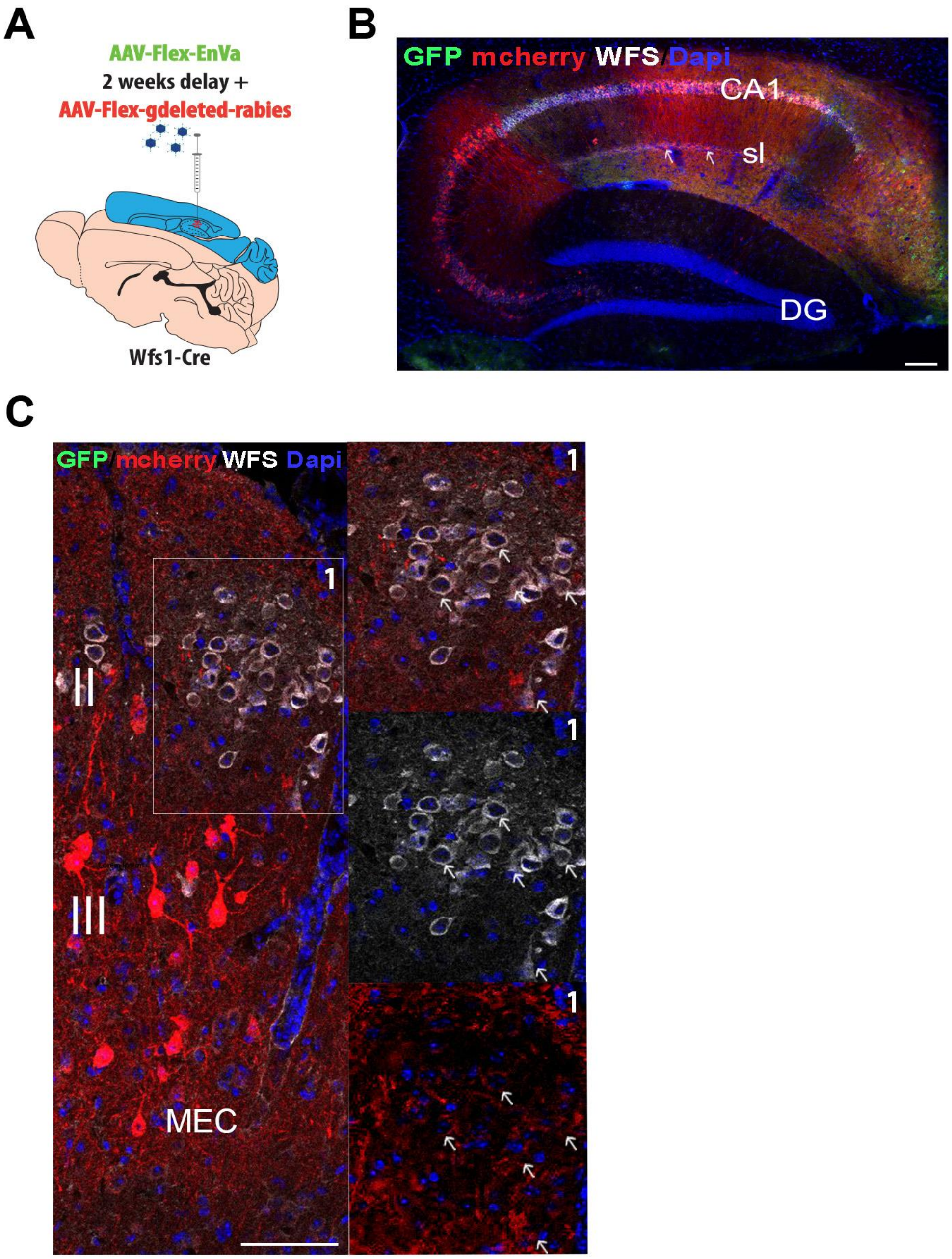

Fig. S15. Characterization of monosynaptic connections of medial entorhinal cortex-to-CA1 Wfs1 neurons using Cre-inducible complementary rabies viral tracing.
(A) Diagram of the mouse brain indicating the location of AAV-Flex-EnVa-GFP injections followed by AAV-Flex-g-deleted-rabies-mCherry into CA1 of Wfs1-Cre mice two weeks post injection. **(B)** Parasagittal section of hippocampus immunostained with anti-GFP (green)/anti-mCherry (red) and anti-Wfs1 (white). **(C)** Parasagittal section of medial entorhinal cortex (MEC) immunostained with anti-GFP (green)/anti-mCherry (red) and anti-Wfs1 (white). Box 1: magnified images of left panel. Scale bars = 100 µm. CA; *Cornu Ammonis*, GFP; green fluorescent protein, MEC; medial entorhinal cortex.

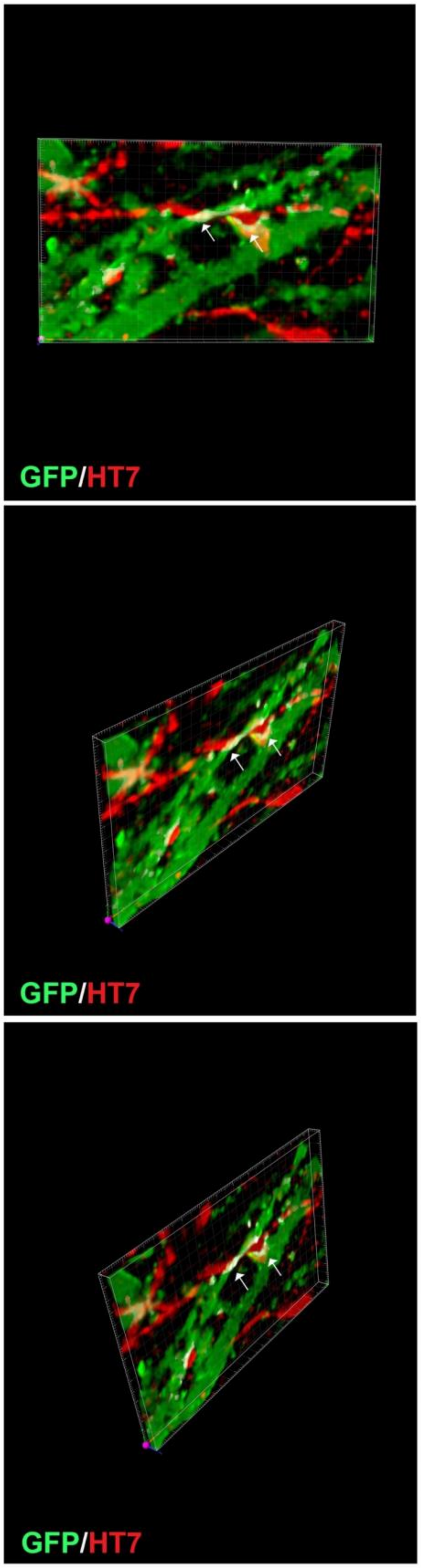

Fig. S16. 3D reconstruction of *stratum lacunosum* connections as shown in Fig. 3D.

Rotated images of the 3D reconstruction of CA1 *stratum lacunosum* region imaged by high-resolution confocal microscopy showing colocalization (white) of tau (red) and GFP (green) signal taken from a parasagittal section of hippocampus of Wfs1-Cre mice injected with AAV2/6-Flex-P301L tau (red) unilaterally into medial entorhinal cortex and AAV2/6-Flex-GFP (green) unilaterally into CA1 and immunostained by anti-HT7/anti-GFP. Scale bars (red/green arrows at the corner) = 2 µm for the major ticks of the 3D frame. See also **Video S2**. CA; *Cornu Ammonis*, GFP; green fluorescent protein.

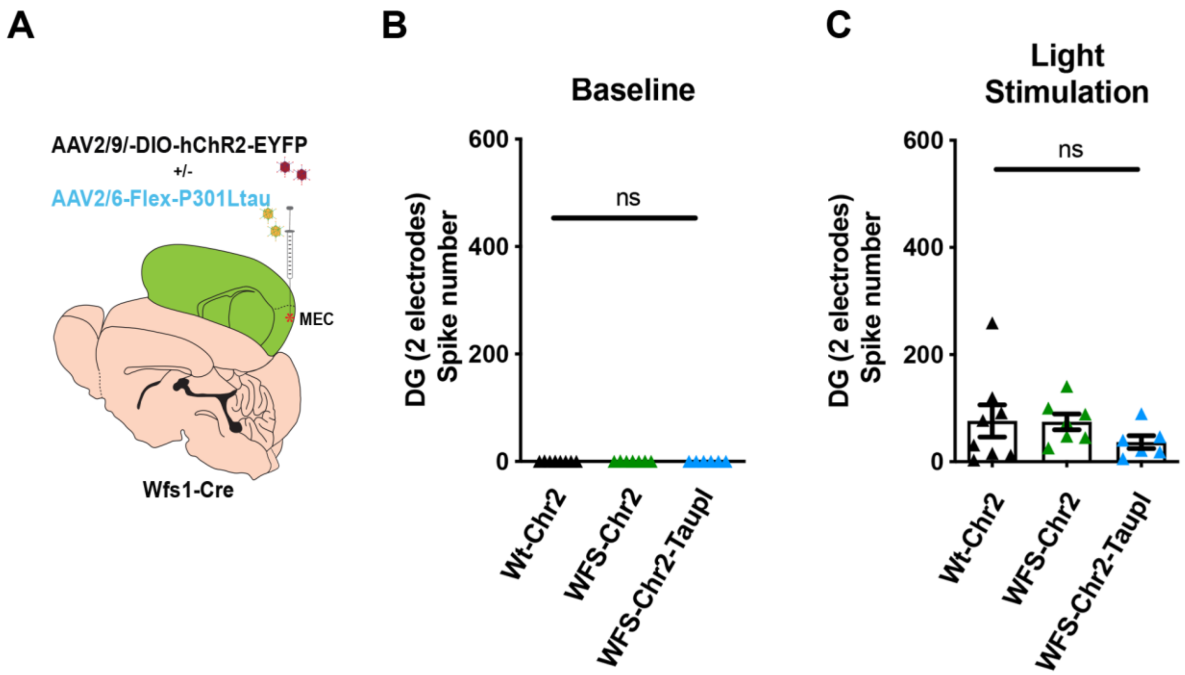

Fig. S17. Effects of tau propagation on CA1 response to optogenetic stimulation in Wfs1-Cre mice.

(**A**) Diagram of the mouse brain indicating the location of AAV2/9-DIO-hChR2-EYFP (humanized channelrhodopsin receptor-2) or AAV2/6-Flex-P301L tau unilateral injections into MEC of Wfs1-Cre mice. (**B-C**) Baseline quantification of the two electrodes located on dentate gyrus (DG), four weeks post injection of a AAV2/9-DIO-hChR2-EYFP with or without injection of Cre-inducible AAV2/6-Flex-P301L tau in wildtype and Wfs1-Cre mice at baseline (ANOVA, tau effect $F_{(2,18)}$=1.48, WT-Chr2 group: n = 8 animals; Wfs1-Chr2 group: n = 7 animals; Wfs-Chr2-tau group: n = 6 animals) **(B)** and after light stimulation activating YFP expressing axonal terminals in HPC (ANOVA, tau effect $F_{(2,18)}$= 0.90, WT-Chr2 group: n = 8 animals; Wfs1-Chr2 group: n = 7 animals; Wfs-Chr2-tau group: n = 6 animals) **(C)**. ns: not significant. See **Table S2** for sample details. CA; *Cornu Ammonis*, ChrR2; channelrhodopsin receptor-2, DG; dentate gyrus, EFYP; enhanced yellow fluorescent protein.

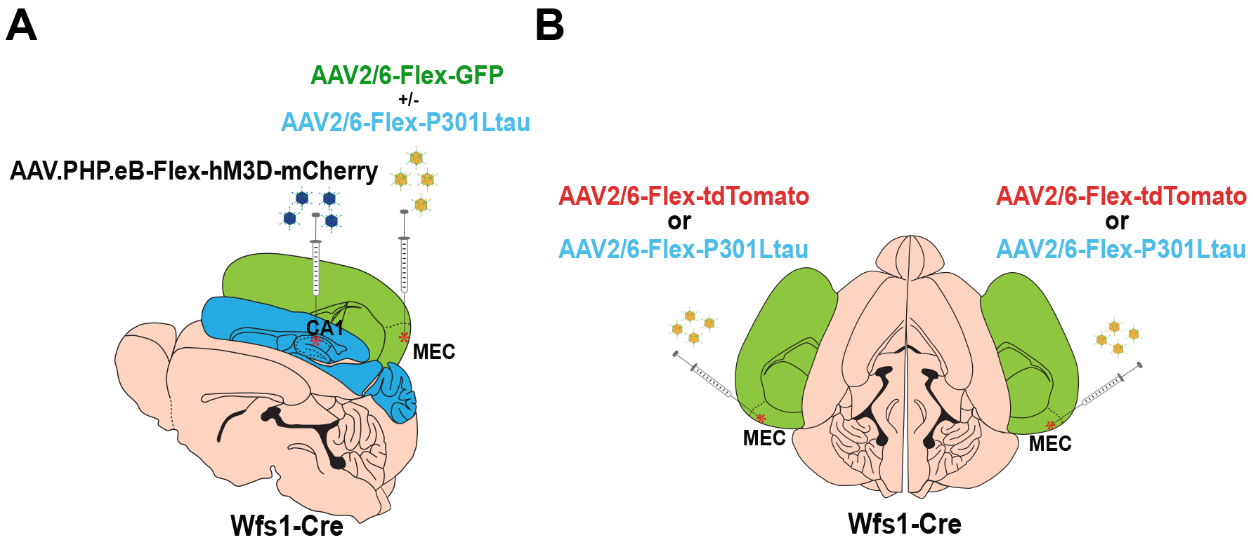

Fig. S18. Diagram of the mouse brain indicating the location of the viral injections into Wfs1-Cre mice.
(A) Diagram of the mouse brain indicating location of AAV.PHP.eB-Flex-hM3D-mCherry (DREADDs activator) unilateral injection into CA1 and AAV2/6-Flex-P301L tau or AAV2/6-Flex-GFP unilateral injection into the medial entorhinal cortex (MEC) of Wfs1-Cre mice. **(B)** Diagram of the mouse brain indicating location of AAV2/6-Flex-tdTomato or AAV2/6-Flex-P301L tau bilateral injections into MEC of Wfs1-Cre mice. MEC, medial entorhinal cortex, DREADD; Designer Receptor Exclusively Activated by Designer Drugs, hM3D; human muscarinic acetylcholine receptor 3 DREADD, GFP; green fluorescent protein.

Video S1. Video depicts 360° rotation of the 3D reconstruction of confocal image stacks of GFP and tdTomato colocalization (white) in *stratum lacunosum* as shown in **Fig. 3B.** (See accompanied Video S1 file)

Video S2. Video depicts 360° rotation of the 3D reconstruction of confocal image stacks of GFP (green) and tau (red) colocalization (white) in *stratum lacunosum* as shown in **Fig. 3D.** (See accompanied Video S2 file)
